# Deletion of Major Endoxylanase Genes in *Fusarium graminearum* Uncovers Distinct Virulence Phenotypes Influenced by the Genetic Background

**DOI:** 10.1111/mpp.70362

**Published:** 2026-10-27

**Authors:** Arianna Panichi, Silvana Odorizzi, Carla Castiglioni, Silvio Tundo, Alessandro Raiola, Francesco Favaron, Wilhelm Schäfer, Luca Sella

**Affiliations:** ^1^ Department of Land, Environment, Agriculture and Forestry (TESAF) University of Padova Legnaro Italy; ^2^ Department of Agronomy, Food, Natural Resources, Animals and Environment (DAFNAE) University of Padova Legnaro Italy; ^3^ Biocenter Klein Flottbek, Molecular Phytopathology and Genetics University of Hamburg Hamburg Germany

**Keywords:** cell wall‐degrading enzymes (CWDEs), deoxynivalenol (DON), necrosis‐inducing factors, plant–pathogen interaction, pleiotropic effects

## Abstract

*Fusarium graminearum*, the main causal agent of Fusarium head blight of wheat, secretes endoxylanases to hydrolyse xylan, a major component of hemicellulose. Mutants of the highly expressed *FGSG_03624* (*xylB*) gene previously showed a disease severity level similar to wild‐type (WT) in wheat spikes. In this study, in contrast, three independent deletion mutants of *FGSG_10999* (*xylA*), a second highly expressed endoxylanase gene, were significantly impaired in disease severity. Surprisingly, a knock‐out mutant of *xylA* in the ΔxylB genetic background (ΔΔxylB+A) exhibited virulence comparable to that of ΔxylB and WT. The ΔxylA mutant shared with ΔΔxylB+A a marked reduction in xylanase activity during in vitro growth, and also with ΔxylB a decreased fungal biomass and deoxynivalenol (DON) accumulation in infected spikes. In contrast to ΔΔxylB+A and ΔxylB, ΔxylA revealed down‐regulation of some endoxylanase and effector genes during spike infection. In addition, when grown on a wheat spike medium, the culture filtrate of ΔxylA showed a significant reduction in total protein secretion and a more severe impairment in necrotizing activity in *Nicotiana benthamiana* infiltrated leaves compared with ΔxylB and ΔΔxylB+A culture filtrates. When a deletion mutant of *xylB* was generated in the ΔxylA genetic background, the ΔΔxylA+B mutant displayed severely reduced virulence, similar to that of the parental ΔxylA. Altogether, these results demonstrate that *xylA* plays a critical role in *F. graminearum* pathogenicity through unexpected pleiotropic, network‐level alterations, as also supported by the partial restoration observed by complementing the *xylA* gene in ΔxylA, highlighting the strong influence of genetic background on phenotypic outcomes.

## Graphical abstract

Deletion of major endoxylanases in *Fusarium graminearum* shows that *xylA* controls virulence via pleiotropic, genetic background‐dependent regulatory effects rather than xylanase activity per se.

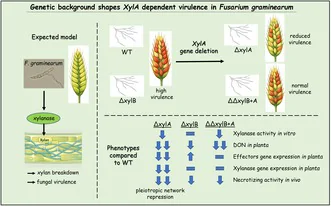

## 1 Introduction

*Fusarium graminearum* is the main causal agent of Fusarium head blight (FHB), a destructive disease of wheat and barley that results in significant yield losses and contamination of grains with mycotoxins, such as deoxynivalenol (DON), posing health risks to humans and animals (Alisaac and Mahlein 2023). To colonize wheat spikes, *F. graminearum* produces cell wall‐degrading enzymes (CWDEs), effectors and toxins (Brown et al. 2012; Zhao et al. 2013). The plant cell wall is one of the first barriers against pathogen invasion (Malinovsky et al. 2014; Wan et al. 2021) and it is mainly constituted of polysaccharides such as hemicellulose, cellulose and pectin (Jung 1995). To degrade the complex polysaccharide network and facilitate cell wall penetration and colonization, *F. graminearum* secretes a wide array of CWDEs, including xylanases, cellulases, pectinases and glucanases (Brown et al. 2012; Yang et al. 2012). Gene expression profiling indicates that most *F. graminearum* CWDE‐encoding genes are up‐regulated during plant infection (Zhao et al. 2013). In addition, infection cushions—multicellular appressoria produced by *F. graminearum* to penetrate the host plant—are enriched in CWDEs (Mentges et al. 2020).

Among CWDEs, xylanases are particularly relevant for their role in hydrolysing xylan, the major hemicellulose component of wheat cell walls. Xylan is mainly composed of a backbone of d‐xylose residues linked by β‐1,4‐bridges variably substituted by other groups (Collins et al. 2005; Hatsch et al. 2006). In particular, endo‐1,4‐β‐d‐xylanases (EC class 3.2.1.8; further referred to as endoxylanases) cleave the β‐1,4 glycosidic bonds in the xylan backbone (Collins et al. 2005). Endoxylanases are mainly grouped into glycoside hydrolase (GH) families 10 (GH10) and 11 (GH11), corresponding to high and low molecular weight xylanases, respectively (Biely et al. 1997; Collins et al. 2005).

The *F. graminearum* genome contains six endoxylanase‐encoding genes—namely *FGSG_03624 (xylB)*, *FGSG_10999 (xylA)*, *FGSG_11487*, *FGSG_06445*, *FGSG_11304* and *FGSG_11258—*that are significantly expressed during wheat spike infection (Sella et al. 2013). Among these, *xylB* and *xylA*, both belonging to the GH11 family, exhibit necrotizing activity that is independent of their catalytic activity (Sella et al. 2013; Tundo et al. 2015) and show the highest expression levels at 3 days post‐inoculation (Sella et al. 2013).

A ΔxylB knock‐out mutant generated in the *F* *. graminearum* PH1 background retains full virulence (Sella et al. 2013). Thus, although *xylB* is highly expressed in planta and possesses necrotizing activity, its absence does not impair successful infection, suggesting that other endoxylanases may compensate for its loss or that the residual xylanolytic activity is sufficient to support effective infection (Sella et al. 2013).

The expression of xylanolytic enzymes in *F. graminearum* is regulated by the Xyr1 transcription factor (Brunner et al. 2007). Deletion of *Xyr1* in the PH1 background strongly reduces xylanase and cellulase activity and severely impairs fungal growth on xylan or wheat cell wall‐derived substrates (Sella et al. 2016). Consistent with its role as a global regulator of xylanolytic gene expression, the Δxyr1 mutant shows a dramatic reduction in the expression of all six *F. graminearum* endoxylanase genes both in vitro and during wheat spike infection, with particularly pronounced effects on *xylA* and *FGSG_06445* genes. Nevertheless, as observed for the ΔxylB mutant, the Δxyr1 mutant retains wild‐type levels of virulence (Sella et al. 2016). These findings suggest that extensive xylanase activity may not be required for *F. graminearum* virulence in wheat.

More recently, deletion of the *xylA* gene in the CS3005 background resulted in significantly reduced virulence, decreased fungal biomass on wheat spikes, and impaired growth on xylose‐containing media, indicating an essential role in xylan utilization and pathogenicity (Sperschneider et al. 2016; Tini et al. 2020). The function of XylA does not seem to be compensated at the transcriptional level by other endoxylanases, such as *FGSG_06445* and *FGSG_11487* (Tini et al. 2020).

These findings raise the question of whether the contribution of individual CWDEs to fungal virulence depends on the genetic context in which gene deletions are introduced. To address these seemingly contradictory findings and gain further insights into the role of endoxylanases in *F. graminearum* pathogenicity, we generated a double mutant (ΔΔxylB+A) by deleting the *xylA* gene in the ΔxylB genetic background. This ΔΔxylB+A mutant was compared with the parental ΔxylB and with three independent ΔxylA mutants, generated in the PH1 background with the same construct used to obtain the double mutant. We characterized these mutants for their (i) enzymatic activities, (ii) virulence on soybean seedlings (used as a model system) and wheat spikes, (iii) fungal biomass and DON production in infected wheat spikes, (iv) gene expression profiles of endoxylanase and selected effector‐encoding genes, and (v) necrosis‐inducing activity.

Finally, we generated a second double mutant (ΔΔxylA+B) using the ΔxylA single mutant as the genetic background and genetically complemented the ΔxylA mutant by introducing the *xylA* gene with native promoter and terminator. This integrated approach allowed us to assess whether the combined deletion of the two highly expressed *F. graminearum* endoxylanase genes leads to compensatory responses or additional pathogenicity defects, and whether these effects depend on the genetic background in which the deletions were generated.

## 2 Results

### 2.1 Production of *F. graminearum* ΔxylA and Double Disruption Mutants Using ΔxylB as Genetic Background

The *xylA* gene was knocked‐out in the *F. graminearum* wild type (WT) and ΔxylB mutant of the PH1 strain through a split‐marker gene replacement cassette. Putative mutants, selected based on antibiotic resistance, were subsequently screened by PCR targeting both the disrupted gene and the antibiotic resistance gene (Figure S1a,b). Three ΔxylA mutants (named ΔxylA_1F, 3F and 5C), obtained from the WT strain, and one double mutant (named ΔΔxylB+A), obtained in the ΔxylB genetic background, were selected and further analysed by PCR using specific primer pairs designed to verify the correct integration of the gene replacement cassette at the target locus (Figure S1c,d). Notably, the ΔxylA_5C single mutant failed to amplify the upstream region of the integration locus, suggesting that an unexpected mutation occurred during the insertion of the resistance cassette (Figure S1c). For all the other mutant strains, both upstream and downstream flanking regions amplified correctly (Figure S1c,d). All ΔxylA single mutants and the double ΔΔxylB+A mutant showed a single integration of the geneticin resistance gene as determined by quantitative PCR (qPCR) (Figure S2).

### 2.2 Characterization of the ΔxylA Single Mutants and ΔΔxylB+A Mutant In Vitro and In Planta

On complete medium agar (CMA; Leslie and Summerell 2006), the ΔxylA_3F and ΔxylA_5C transformants showed significantly reduced radial growth compared to WT, ΔxylB, and ΔΔxylB+A mutants (*p* < 0.05). The ΔxylA_1F mutant showed a slight, but not statistically significant, reduction in radial growth (Figure S3a). Because CMA does not contain xylan, these results suggest that the reduced growth of the ΔxylA mutants is likely unrelated to xylan metabolism.

The ΔxylA mutants exhibited a distinct phenotype compared to the WT (Figure S3b). Both ΔxylA_1F and ΔxylA_3F displayed a zonal growth pattern characterized by a bright yellow coloration in the mature centre, which was larger than the white peripheral mycelium. In the ΔxylA_5C mutant, the central pigmented area was strongly reduced in size, resulting in a predominantly white mycelium. In contrast, both the ΔxylB single mutant and the ΔΔxylB+A double mutant maintained the red pigmentation of the WT mycelium in their mature centres (Figure S3b).

Enzymatic activities produced by WT and mutant strains were measured after 4 days of growth in Szécsi liquid medium supplemented with xylan, carboxymethyl cellulose (CMC) or apple pectin as the sole carbon source. Total xylanase, cellulase and polygalacturonase activities in the supernatants were quantified and normalized to fungal dry weight (Figure 1).

**FIGURE 1:**
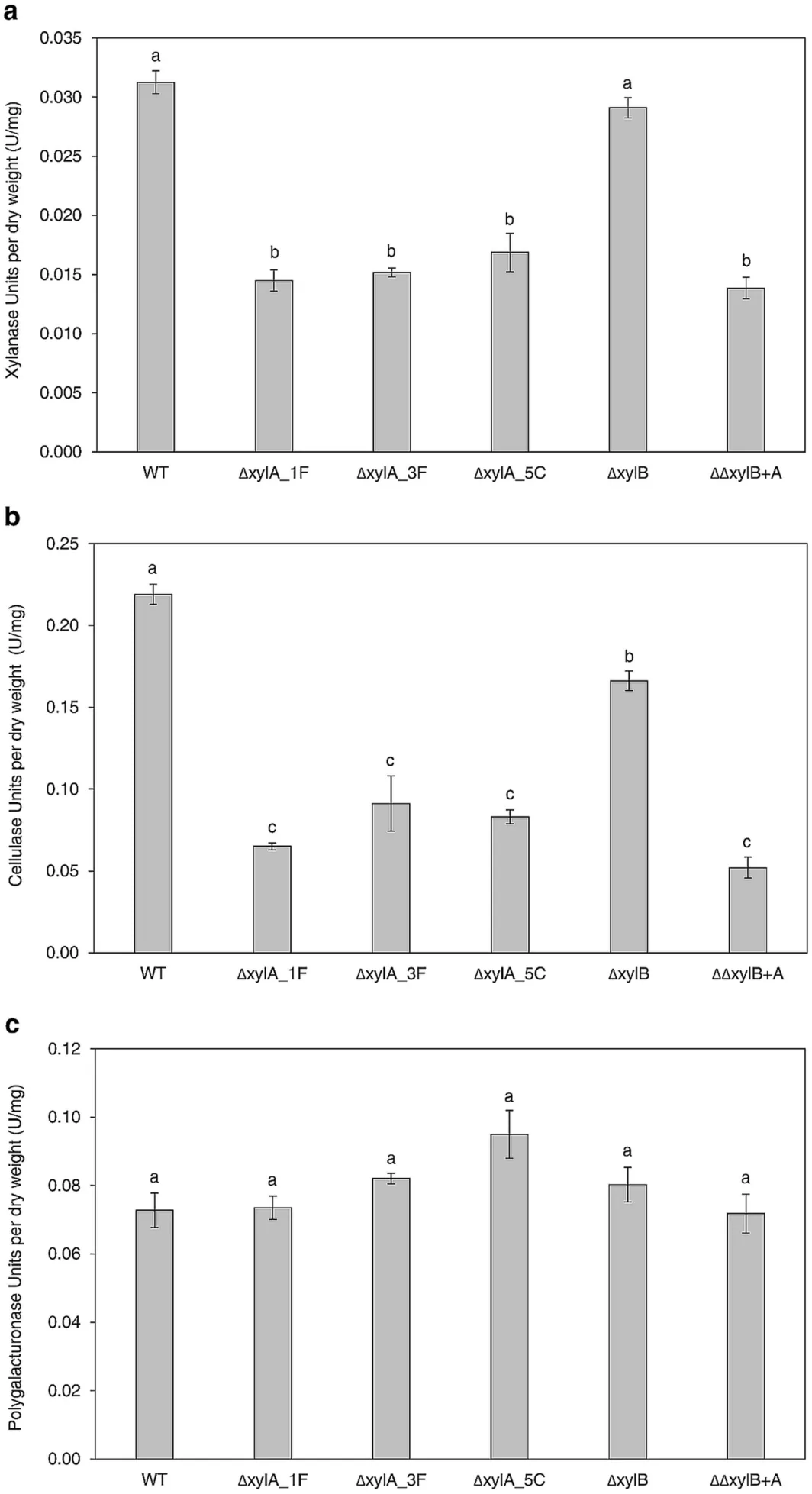
Total xylanase (a), cellulase (b), and polygalacturonase (c) activities secreted by *Fusarium graminearum* wild‐type (WT) and mutant strains and normalized per dry weight. Culture filtrates were collected after 4 days of growth in Szécsi liquid medium supplemented with beechwood xylan, carboxymethyl cellulose or apple pectin as sole carbon source and assayed. Total xylanase activity was measured using the dinitrosalicylic acid (DNSA) method. One xylanase unit was defined as the amount of enzyme required to release 1 μmol of xylose under the assay conditions. Cellulase and polygalacturonase activities were determined by viscosimetry. One enzymatic unit corresponds to the amount of enzyme that decreases by 10% the initial relative viscosity of the substrate within 40 min. Three independent experiments were performed. Data are presented as mean ± standard error. Statistical analyses were conducted using one‐way ANOVA followed by Tukey–Kramer post hoc test. Different letters indicate significant differences at *p* < 0.05.

Deletion of the *xylA* gene significantly affected total xylanase activity, resulting in an approximately 50% reduction in all three ΔxylA single mutants and in the ΔΔxylB+A double mutant compared with the parental strain. On the other hand, the ΔxylB mutant showed no significant reduction in xylanase activity compared to the WT strain (Figure 1a). Cellulase activity was significantly reduced in all the mutant strains (Figure 1b), with reductions around 60% in the ΔxylA single mutants, 75% in the ΔΔxylB+A double mutant, and 25% in the ΔxylB mutant compared to the WT strain. No significant changes in polygalacturonase activity were observed in any of the mutants in comparison to the WT (Figure 1c). Interestingly, the most evident difference in fungal dry weight was observed when the strains were grown on cellulase‐inducing medium (Szécsi liquid medium supplemented with CMC): the ΔxylB mutant and mostly the ΔΔxylB+A double mutant produced significantly more dry weight compared to WT, approximately 50% and 60% more, respectively, while the dry weight of WT and ΔxylA single mutants was similar (Figure S4b).

Virulence tests were conducted on both soybean seedlings and wheat spikes. Soybean seeds were inoculated with germinated conidia and, after 7 days, the Disease Index was calculated. The results indicate that only the ΔxylA single mutants exhibited a significant reduction in virulence. In contrast, the ΔxylB single mutant, the ΔΔxylB+A double mutant, and an ectopic mutant (ΔΔECT), included for comparison and corresponding to a Δ*xylB* mutant, showed a virulence statistically comparable to that of the WT strain (Figure 2a).

**FIGURE 2:**
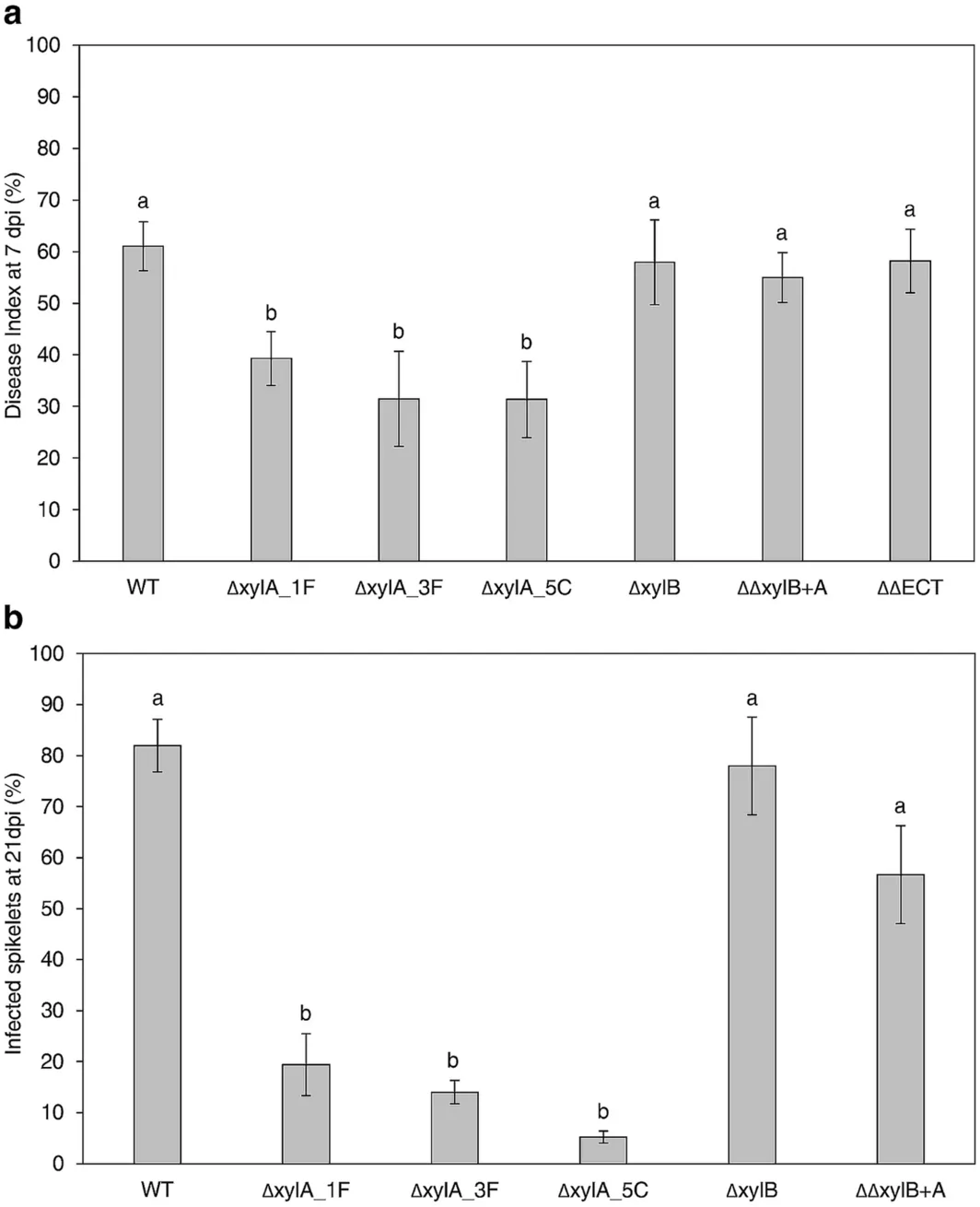
Virulence of *Fusarium graminearum* wild‐type (WT) and mutant strains on soybean seedlings (a) and wheat spikes (b). Soybean seeds were inoculated with a suspension of 24‐h germinated conidia and incubated at 25°C in a humid chamber. Disease Index was calculated at 7 days post‐inoculation (dpi) as the ratio between lesion length and total seedling length × 100. *Triticum aestivum* ‘Bobwhite’ spikes were point‐inoculated at anthesis with 2000 conidia. Disease symptoms were evaluated at 21 dpi by counting the percentage of infected spikelets. Data are the mean ± standard error of at least five independent experiments for soybean (each performed inoculating 20 seeds per fungal strain) and three independent experiments for wheat (each performed inoculating at least seven spikes per fungal strain). Statistical analyses were conducted using one‐way ANOVA followed by the Tukey–Kramer post hoc test. Different letters indicate significant differences at *p* < 0.05.

For wheat, spikes were point inoculated at anthesis and disease symptoms were evaluated 21 days post‐inoculation (dpi). All three ΔxylA single mutants showed significantly reduced virulence compared to both WT and the ΔxylB mutant, with a 75%–94% decrease in infected spikelets relative to WT. No statistically significant differences were detected among the three ΔxylA strains. The ΔΔxylB+A double mutant exhibited a slight, but not statistically significant, reduction in virulence compared to both WT and the ΔxylB mutant (Figure 2b). These results are consistent across both host plants and indicate that the contribution of *xylA* to fungal virulence is strongly dependent on genetic background.

The infected wheat spikes were analysed for fungal biomass and DON contamination (Figure 3). Fungal biomass was assessed through qPCR using specific primers for the fungal *TRI‐6* gene and using as template total DNA extracted from infected spikes. All knock‐out mutants showed a significant reduction in biomass compared to the WT strain (Figure 3a). Specifically, the ΔxylA mutant strains exhibited an 80% reduction in biomass, the ΔxylB mutant a 70% reduction, and the ΔΔxylB+A double mutant a 55% reduction. DON quantification revealed a substantial decrease in mycotoxin contamination in all the mutants (Figure 3b), with the lowest levels of DON observed in the mutants deleted of the *xylA* gene (approximately 6–11 ppm for the ΔxylA mutants and 10 ppm for the ΔΔxylB+A mutant). The ΔxylB mutant displayed a 75% reduction in DON production compared to the WT strain (approximately 38 ppm for the ΔxylB mutant and 155 ppm for the WT).

**FIGURE 3:**
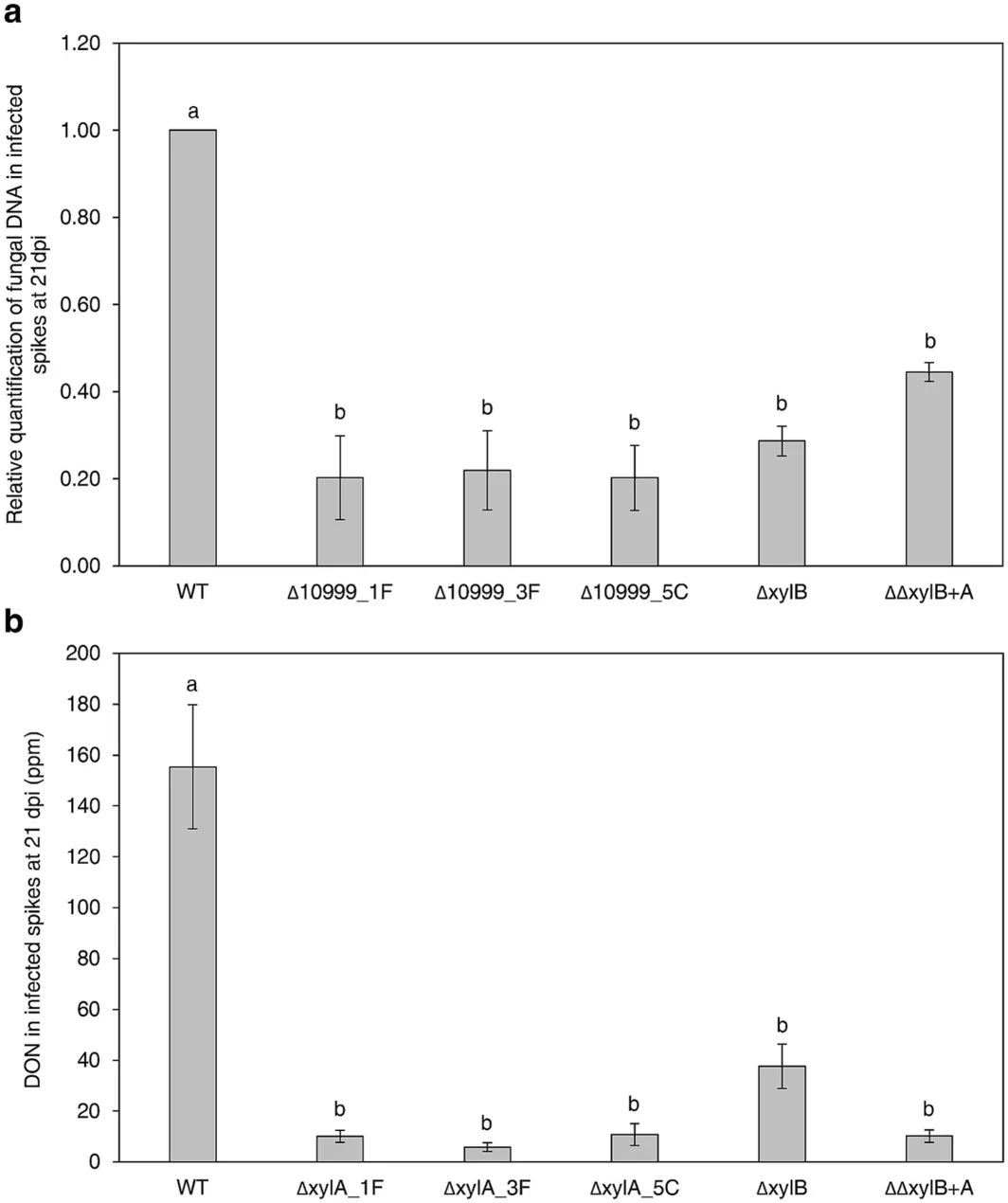
Quantification of fungal biomass and deoxynivalenol (DON) contamination in infected *Triticum aestivum* spikes. Spikes collected at 21 days post‐inoculation (dpi) were ground into fine powder and used for (a) fungal biomass quantification by quantitative PCR, and (b) deoxynivalenol (DON) quantification by ELISA. Data are presented as average values of three independent experiments. Bars represent standard errors. Dunnett's multiple comparisons test was applied for fungal biomass analysis. ANOVA followed by Tukey's post hoc test was used for DON quantification. Different letters indicate significant differences at *p* < 0.05. WT, wild‐type strain.

### 2.3 Expression Analysis of *F. graminearum* Genes Encoding Endoxylanases, Secreted Effector Proteins and Enzymes Involved in Biosynthesis of Secondary Metabolites

Expression analysis in wheat spikes of the six *F. graminearum* endoxylanase‐encoding genes was carried out at 3 dpi to assess whether deletion of *xylA* or *xylB* affects the transcriptional regulation of the other endoxylanase genes. The ΔxylA_1F, ΔxylB_3F, ΔxylB, ΔΔxylB+A, and WT strains were included in the analysis. No significant differences in the expression levels of the endoxylanase genes were observed in either the ΔxylB mutants or the ΔΔxylB+A double mutant compared to WT, whose gene expression was normalized to 1 (Figure 4a). In contrast, the ΔxylA mutants exhibited a down‐regulation of the *FGSG_06445* and, notably, of the *xylB* gene. In addition, ΔxylA_1F, one of the two ΔxylA mutants analysed, showed a down‐regulation also of the *FGSG_11304* gene.

**FIGURE 4:**
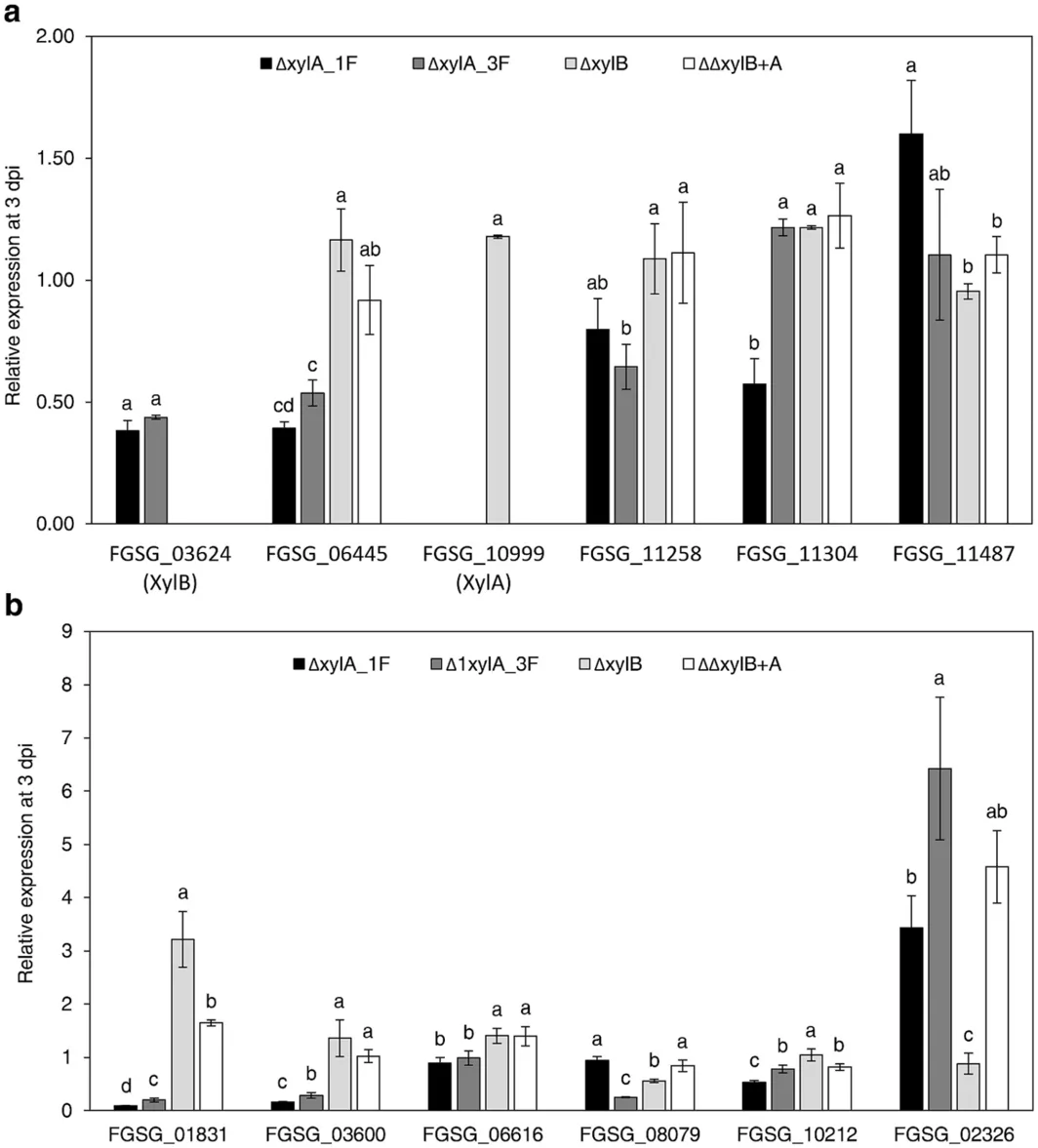
Expression analysis of *Fusarium graminearum* genes in *Triticum aestivum* spikes infected with wild‐type and mutant strains at 3 days post‐inoculation (dpi). (a) Expression analysis of endoxylanase‐encoding genes. (b) Expression analysis of genes encoding effector proteins (FGSG_01831, FGSG_03600, FGSG_10212), enzymes involved in butenolide (FGSG_08079) and aurofusarin (FGSG_02326, AurJ) biosynthesis, and glucan 1,3‐β‐glucosidase (FGSG_06616). Total RNA was extracted from spikelets point‐inoculated at anthesis with 2000 conidia of wild‐type or mutant strains. Reverse transcription‐quantitative PCR was performed using the *F. graminearum β‐tubulin* gene as a housekeeping reference gene. Relative expression levels were analysed using the Rotor‐Gene v. 2.0.3.2 software (Qiagen) and the REST (Relative Expression Software) tool. Gene expression levels in the wild‐type strain were arbitrarily set to 1. Data represent the mean of at least three independent experiments. Error bars indicate standard error. Statistical analyses were conducted using one‐way ANOVA followed by Tukey–Kramer post hoc test. Different letters indicate significant differences at *p* < 0.05.

Given that the XylB and XylA endoxylanases are known to act as necrotizing factors, we analysed whether their deletion might lead to compensatory changes in the expression of other *F. graminearum* genes encoding proteins potentially involved in pathogenicity or in the biosynthesis of toxic secondary metabolites. In particular, we selected the genes *FGSG_01831* (Hao et al. 2020) and *FGSG_03600* (Jin et al. 2024), encoding two necrotizing virulence‐related proteins, the gene *FGSG_10212* encoding the cerato‐platanin protein FgCPP1 (Quarantin et al. 2019), and the genes *FGSG_08079* and *FGSG_02326* involved in the biosynthesis of the mycotoxins butenolide (Harris et al. 2007) and aurofusarin, respectively. We also included in the gene expression analysis a gene encoding glucan 1,3‐β‐glucosidase (*FGSG_06616*), an enzyme that breaks down β‐1,3‐glucans, key components of fungal cell walls, playing a role in cell wall remodelling and potentially affecting fungal growth and virulence (Yang et al. 2012). Gene expression analysis was conducted at 3 dpi.

Deletion of the *xylB* gene led to up‐regulation of the virulence‐related *FGSG_01831* effector gene compared to the WT strain. In contrast, the expression of this gene was strongly down‐regulated in both ΔxylA single mutant strains analysed compared to the WT strain. These mutants also exhibited reduced expression of the *FGSG_03600* effector‐encoding gene and a slight decrease in the expression of the cerato‐platanin gene *FGSG_10212* (Figure 4b).

The expression of genes involved in mycotoxin biosynthesis also varied among mutants. The *FGSG_08079* gene, involved in butanolide biosynthesis, displayed expression levels comparable to the WT in both ΔxylA_1F and ΔΔxylB+A, while the ΔxylA_3F mutant showed down‐regulation (Figure 4b). The expression analysis also revealed the up‐regulation of *AurJ* (*FGSG_02326*), involved in aurofusarin biosynthesis, in both ΔxylA_1F and the ΔxylA_3F single mutants, as well as in the ΔΔxylB+A double mutant, but not in the single mutant lacking *xylB* (Figure 4b). Regarding the *FGSG_06616* gene, encoding a glucan 1,3‐β‐glucosidase, both ΔxylA single mutants showed expression levels comparable to the WT, while the ΔxylB single mutant and the ΔΔxylB+A double mutant displayed a slight up‐regulation.

### 2.4 Necrosis Assays in *Nicotiana benthamiana* Leaves

The necrotizing activity of WT and xylanase mutant strains was evaluated on *N. benthamiana* leaves. Equal volumes of fungal culture filtrates, obtained from Szécsi liquid medium supplemented with wheat spikelets collected at the flowering stage, were infiltrated into leaves, and necrotic areas were measured 4 days post‐infiltration. A negative control (CT–), consisting of Szécsi liquid medium supplemented with spikelets but not inoculated with *F. graminearum* conidia, was also included. The results indicated a reduced necrosis‐inducing activity in all the mutants' culture filtrates compared to that of the WT. Notably, the ΔxylA single mutants' filtrates exhibited the smallest necrotic area, with a 78% reduction compared to the WT, while the ΔxylB single mutant filtrate and that of the ΔΔxylB+A double mutant displayed comparable levels of necrotic area, showing a 45% and 48% reduction compared to the WT, respectively (Figures 5 and S5a). To determine whether the reduced necrotizing activity could be associated with protein secretion, the total protein concentration of culture filtrates was quantified using the Bradford assay. The ΔxylA mutants showed markedly lower concentrations of secreted proteins than WT, ΔxylB and ΔΔxylB+A strains (Figure S5). The observed necrosis was not due to DON, because its level in all the culture filtrates was below the detection limit (LOD = 0.05 ppm; data not shown).

**FIGURE 5:**
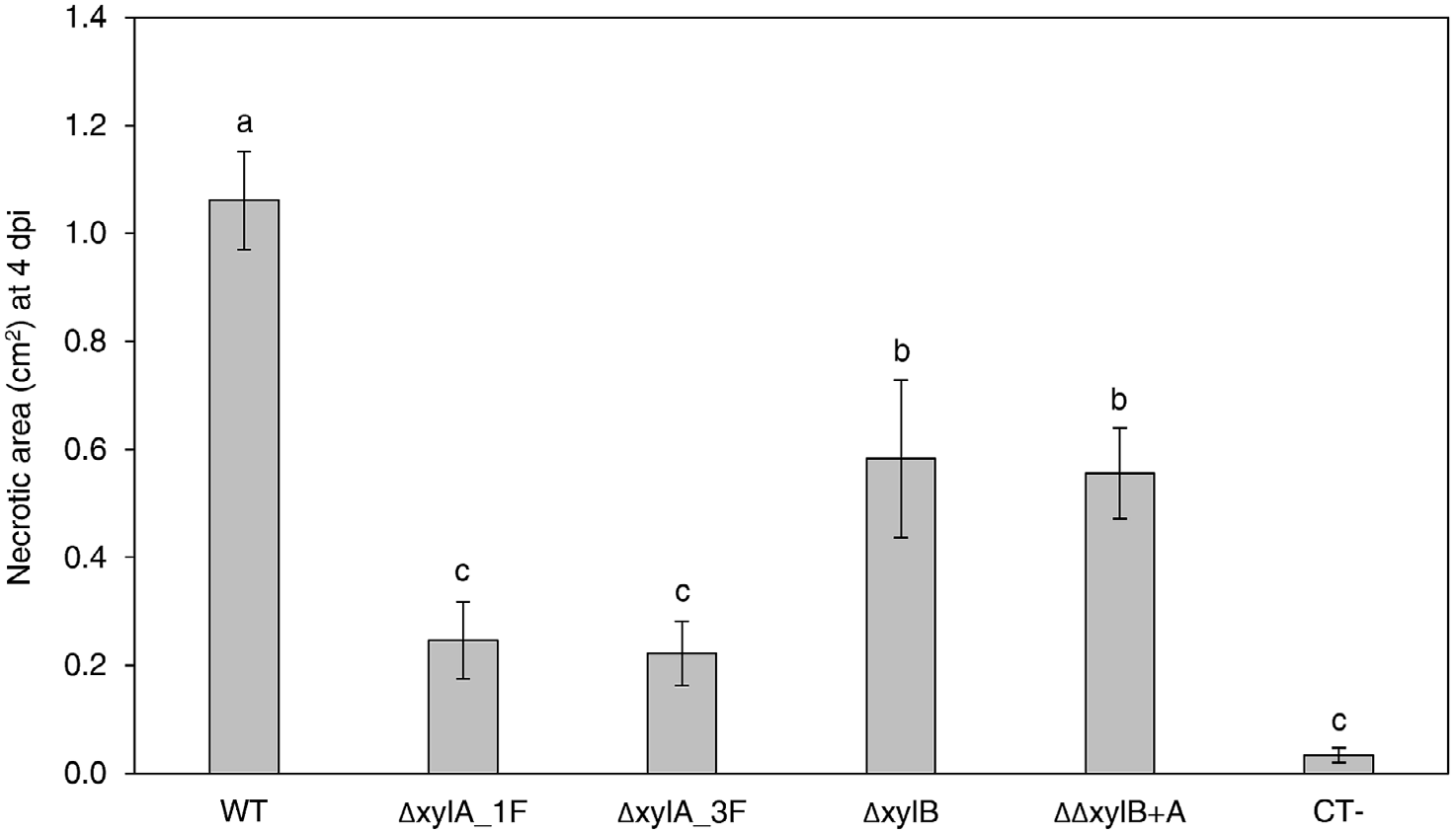
Quantification of necrotic areas in *Nicotiana* *benthamiana* leaves infiltrated with fungal culture filtrates obtained growing conidia in Szécsi liquid medium supplemented with wheat spikelets at flowering stage. One hundred microlitres of culture filtrate was infiltrated into *N. benthamiana* ‘SR1’ leaves. A negative control (CT−), consisting of Szécsi liquid medium supplemented with wheat spikelets, was included. Necrotic areas were measured at 4 days post‐infiltration (dpi). Data represent the mean of at least three independent experiments, each involving four infiltrations of at least three leaves. Error bars indicate standard error. Statistical analyses were conducted using one‐way ANOVA followed by Tukey–Kramer post hoc test. Different letters indicate significant differences at *p* < 0.05. WT: Wild‐type strain.

### 2.5 Generation of a Double Mutant Using the ΔxylA Mutant as the Parental Strain and Virulence Assays on Soybean and Wheat Spikes

The ΔΔxylB+A double mutant, used in the previous experiments, was created by disrupting the *xylA* gene in the ΔxylB genetic background. Surprisingly, the ΔΔxylB+A double mutant and the ΔxylB single mutant exhibited similar phenotypes and virulence levels (Figure S6). This result contrasts with the significant changes observed when the *xylA* gene was disrupted in the WT background. To explain this unexpected outcome, we hypothesized that the initial *xylB* gene disruption might have altered the genetic or physiological state of the recipient strain, influencing the effects of subsequent deletions. Consequently, the order in which these two mutations were introduced may have significantly impacted the features of the resulting strain.

To test this hypothesis, we generated a new double mutant (named ΔΔxylA+B) using the ΔxylA_3F single mutant as genetic background and the hygromycin resistance cassette previously used by Sella et al. (2013) for disrupting the *xylB* gene. The resulting ΔΔxylA+B double mutant was confirmed by PCR (Figure S7) and contained a single integration of the hygromycin gene (Figure S2).

Compared to the ΔxylA single mutants, the ΔΔxylA+B mutant exhibited a similar colony phenotype, with a bright yellow coloration in the mature centre, but reduced radial growth (Figure S3). The ΔxylA_3F mutant, the two double mutants ΔΔxylA+B and ΔΔxylB+A, and the WT strain were subsequently tested for virulence on soybean and wheat plants.

The soybean seedlings infection assay revealed that the virulence of the newly developed ΔΔxylA+B double mutant was comparable to that of the parental ΔxylA_3F mutant. Importantly, its virulence was significantly lower than that of the previously characterized ΔΔxylB+A mutant, whose virulence was not significantly different from that of the WT (Figure 6a).

**FIGURE 6:**
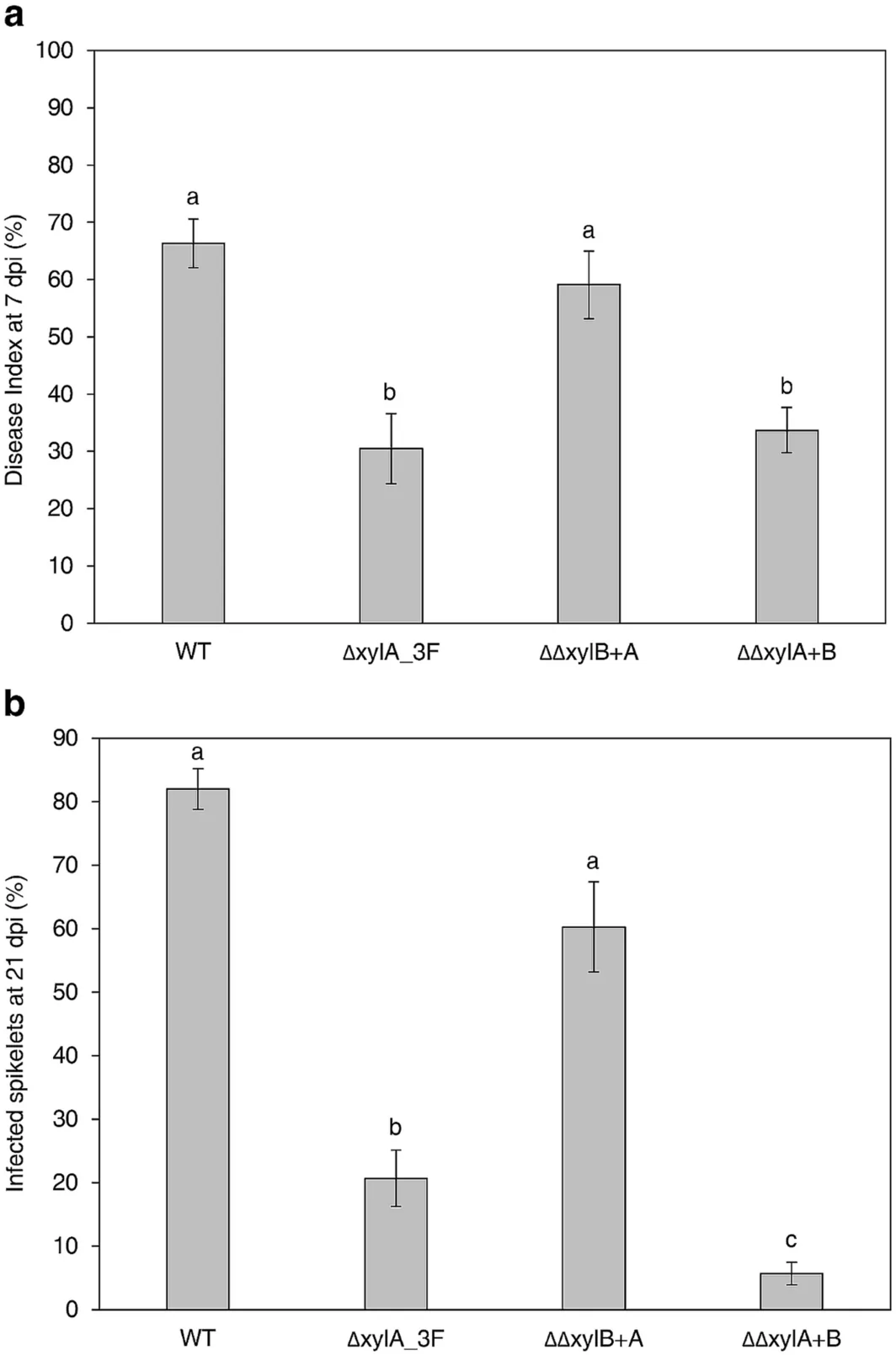
Virulence assays on soybean seedlings (a) and wheat spikes (b). Pre‐germinated conidia of wild‐type (WT) and mutant strains were used to inoculate soybean seedlings, and disease index was evaluated at 7 days post‐inoculation (dpi). *Triticum aestivum* ‘Bobwhite’ spikes were point inoculated at anthesis with 2000 conidia, and disease symptoms were evaluated at 21 dpi by counting the percentage of infected spikelets. Data are presented as mean ± standard error of at least three independent experiments, each performed inoculating 20 soybean seeds or at least seven wheat spikes per fungal strain. Statistical analyses were conducted using one‐way ANOVA followed by Tukey–Kramer post hoc test. Different letters indicate significant differences at *p* < 0.05.

Notably, the infection assay on wheat spikes ( *Triticum aestivum* ‘Bobwhite’) revealed a pronounced decrease (−93%) in the percentage of infected spikelets in the ΔΔxylA+B double mutant compared to WT, while the reduction in the ΔΔxylB+A double mutant was not significantly different from the WT. The reduction in infected spikelets observed with the ΔΔxylA+B double mutant was significantly lower than that of the ΔxylA_3F single mutant strain (Figure 6b).

### 2.6 Complementation of the *xylA* Gene in the ΔxylA Mutant

Two selected complemented strains, obtained by reintroducing the *xylA* gene into the ΔxylA_3F genetic background (Figure S8a,b), were characterized. The *xylA* gene replaced the geneticin resistance gene originally used to delete *xylA* in both C2.1 and C3.8 strains (Figure S8c), the former containing two additional copies of *xylA* (Figure S8d). *XylA* gene expression was restored in both complemented strains (Figure S8e). When grown on CMA, C2.1 and C3.8 displayed partial restoration of fungal growth compared to ΔxylA_3F and WT strains, and almost restored pigmentation, similar to that of the WT (Figure S9a,b). When grown on xylan, both complemented strains showed restored xylanase activity, while ΔxylA_3F produced ~40% less xylanase than the WT (Figure S9c).

Inoculation assays on *T. aestivum* ‘Bobwhite’ spikes showed that both complemented strains exhibited an intermediate phenotype between the WT and the ΔxylA_3F mutant, indicating a partial restoration of virulence. In particular, C2.1 and C3.8 exhibited approximately 50% fewer infected spikelets than the WT, whereas the ΔxylA_3F mutant showed a reduction of approximately 75% (Figure S10a). However, the increase in the percentage of infected spikelets observed in the complemented strains relative to ΔxylA_3F was not statistically significant (Figure S10a). The biomass of C2.1, C3.8 and ΔxylA_3F in infected wheat spikes was found to be proportionally reduced compared to the WT strain (Figure S10b). Interestingly, DON quantification revealed that both complemented strains were still impaired in DON production (approximately 7–8 ppm), similar to the ΔxylA_3F mutant (approximately 3 ppm) (Figure S10c).

## 3 Discussion

Fungal endoxylanases have been shown to be key virulence factors in numerous pathosystems, functioning primarily as CWDEs that break down hemicellulose to facilitate host colonization, but also acting as necrotizing factors in plant cells (Brito et al. 2006; Cui et al. 2024; García et al. 2017; Lai and Liou 2018; Liu et al. 2024; Ma et al. 2024; Moreno‐Sánchez et al. 2021; Shabbir et al. 2022; Yu et al. 2018; Wang et al. 2021). In *F. graminearum*, the endoxylanase‐encoding genes *xylB* and *xylA* are the most highly expressed during early wheat infection (Sella et al. 2013). Despite their overlapping functions, our findings based on the characterization of knock‐out mutants reveal distinct impacts of these two genes on enzymatic activity, virulence, and on the regulation of other virulence‐related genes.

Deletion mutants of the *xylA* gene generated in the PH1 strain background consistently showed a marked reduction in virulence on soybean and wheat, as well as a substantial reduction of biomass accumulation and DON production in infected spikes, confirming previous results obtained by Tini et al. (2020) using a different WT strain. Two of these mutants (ΔxylA_1F, ΔxylA_3F) were further characterized and showed reduced necrosis‐inducing activity, probably reflecting both a lower abundance of secreted proteins and qualitative changes in the secreted necrotizing factors, and significant down‐regulation of genes encoding other endoxylanases (namely *xylB* and *FGSG_06445*) and effectors involved in pathogenicity, confirming and extending previous findings (Tini et al. 2020). In particular, the ΔxylA mutant strains showed a strong down‐regulation of the *FGSG_01831* gene, which encodes a small secreted effector previously shown to enhance *F. graminearum* aggressiveness during wheat infection (Brown et al. 2012), and of the *FGSG_03600* gene, which encodes a nuclear effector protein that targets the host plant's nucleus to suppress immune responses and promote fungal virulence (Jin et al. 2024).

In contrast, the ΔxylB mutant (formerly named as ΔXyl11; Sella et al. 2013) did not exhibit any significant reduction in disease severity on wheat or soybean, consistent with previous findings by Sella et al. (2013), who tested three independent ΔxylB mutant strains, including the one analysed here, and found WT‐like virulence on wheat. Nevertheless, following wheat inoculation with the ΔxylB mutant, a substantial and unexpected decrease in fungal biomass and DON accumulation in wheat spikes was detected, suggesting that the XylB endoxylanase is necessary to support full host colonization. Moreover, deletion of the *xylB* gene triggered a compensatory response, including up‐regulation of the *FGSG_01831* effector gene, which, by contrast, was down‐regulated in the ΔxylA mutant. This result is consistent with the maintenance of the infection potential of this strain and with the known ability of fungal pathogens to adjust their virulence repertoire under stress conditions (Mentges et al. 2020).

Another feature that distinguishes the ΔxylA mutant strains from the ΔxylB mutant is their reduced in vitro xylanase and cellulase activity. However, these differences in enzymatic activity do not account for the contrasting virulence phenotypes observed between the ΔxylA and ΔxylB mutants. This lack of correlation was demonstrated by the characterization of the ΔΔxylB+A double mutant generated by deleting *xylA* in the ΔxylB background. This ΔΔxylB+A mutant displayed reduced xylanase and cellulase activity in vitro as well as reduced DON accumulation in planta similar to the ΔxylA mutant strains, yet retaining a virulence phenotype comparable to that of the ΔxylB mutant and the WT (Figure S6). This evidence supports the hypothesis that deletion of *xylB* may have triggered transcriptional or biochemical adaptations that persist in the ΔΔxylB+A double mutant, thereby mitigating the effects of the subsequent *xylA* deletion. This interpretation is consistent with our observation that the *FGSG_01831* effector gene, which was transcriptionally up‐regulated in the ΔxylB mutant, maintained WT expression levels in the ΔΔxylB+A mutant. Similarly, the *FGSG_03600* effector gene and the *FGSG_06445* endoxylanase gene displayed in the ΔΔxylB+A mutant expression levels comparable to those of the WT and ΔxylB mutant (Figure S6).

Interestingly, the ΔΔxylB+A mutant accumulated markedly lower DON levels in infected spikes. However, the mycotoxin was quantified from whole‐spike extracts and therefore may not reflect local DON concentrations at the infection front (e.g., rachis node). A reduced total DON accumulation may not necessarily alter the progression of the fungus through the rachis. As previously observed, the relationship between visual symptoms and DON content deteriorates at higher infection levels (Lemmens et al. 2004).

The ΔΔxylB+A mutant exhibited higher growth than ΔxylA on CMC‐containing medium and produced more biomass in the infected wheat tissue, even though this difference was not significant. Despite having measured in vitro a ΔΔxylB+A cellulase activity comparable to that of the ΔxylA mutant, it should be noted that the viscosimetric assay used in this work, with CMC as substrate, primarily detects endoglucanase activity rather than exoglucanases, due to the soluble and amorphous nature of CMC (Sohail et al. 2009). Considering both in vitro and in vivo growth data, the ΔΔxylB+A mutant may still compensate for the loss of the two endoxylanases by up‐regulating other cellulose‐active enzymatic activities, like lytic polysaccharide monooxygenases (LPMO) or β‐glucosidases, not measurable in our viscosimetric assay.

Given the unexpected similarity between ΔΔxylB+A and ΔxylB mutants, we generated the ΔΔxylA+B mutant by deleting the *xylB* gene in the ΔxylA background, to assess whether reversing the order of gene deletions would result in a different phenotype. The ΔΔxylA+B double mutant displayed a severely reduced virulence phenotype, comparable to ΔxylA on soybean seedlings and even stronger on wheat spikes. This indicates that once *xylA* is removed, the deletion of *xylB* does not seem to induce evident compensatory mechanisms. Unlike ΔΔxylB+A, which phenotypically resembles ΔxylB, although exhibiting clear biochemical impairment, the ΔΔxylA+B double mutant mirrored ΔxylA at phenotypic level, with a bright yellow coloration in the mature centre of the fungal colony and a reduced growth on complete medium. This outcome, despite identical genetic deletions, emphasizes the importance of genetic background and deletion order and suggests that pleiotropic effects, driven by the sequence of genetic modifications, can shape the adaptive capacity of the fungus. Notably, the slightly reduced growth displayed by the ΔxylA mutant on complete medium possibly indicates defective nutrient uptake, which could further impair its ability to exploit plant tissues for nutrition, and support the hypothesis that the virulence defect of ΔxylA arises from pleiotropic network‐level effects. The partial, although not statistically significant, restoration of virulence exhibited by the complemented strains on wheat spikes, despite complete recovery of xylanase activity and almost restored pigmentation and fungal growth in vitro, further supports this interpretation. Moreover, DON production remained strongly impaired in both complemented strains, suggesting that this phenotype is not readily reversed by restoration of *xylA* expression and enzymatic activity and may thus reflect persistent pleiotropic changes established following *xylA* disruption.

Although the molecular basis of this phenomenon remains unknown, and additional studies are required to determine whether it reflects regulatory adaptation or other changes associated with the genetic background, our interpretation is supported by studies in model organisms like *Saccharomyces cerevisiae* and *Escherichia coli* , where the phenotypic impact of mutations often depends on the genetic context and interaction between multiple traits governed by shared pathways (Batarseh et al. 2023; Chandler et al. 2014; Costanzo et al. 2010). In *Pseudomonas* spp., fitness effects of resistance mutations vary across strain backgrounds (Vogwill et al. 2016), and in *Cryptococcus neoformans* deletion of the same gene causes divergent outcomes depending on strain‐specific network regulation (Rhodes et al. 2017). Similar findings have been reported in *Neurospora crassa*, where deletion of genes involved in calcium signalling resulted in contrasting phenotypes depending on the combination: while some double mutants showed severe growth and fertility defects, others phenocopied the WT, highlighting compensatory genetic interactions (Barman and Tamuli 2017). Although whether similar mechanisms operate in *F. graminearum* remains to be established, these examples reinforce the idea that the effect of gene deletions in *F. graminearum*, especially those affecting virulence, could be shaped not only by gene function but also by the fungal ability to reprogramme regulatory and metabolic responses. In summary, our findings confirm that *xylA* is a critical virulence factor in *F. graminearum*. However, its contribution to pathogenicity cannot be attributed solely to its enzymatic or necrosis‐inducing activity. Instead, this gene appears to function as a regulatory hub within the network of CWDEs and effectors, with phenotypic outcomes emerging from context‐dependent interactions rather than from the loss of a single function. The strong virulence reduction observed in the ΔxylA mutant appears to stem from pleiotropic effects, particularly the down‐regulation of other endoxylanase genes (including *xylB*), the dramatic repression of two effector genes, and the reduced necrotizing activity compared to the ΔxylB and ΔΔxylB+A mutants and to the WT. This hypothesis is consistent with the phenotype of the complemented strains, whose virulence was only partially restored.

Interestingly, the ΔΔxylB+A double mutant lacking the *xylA* gene in the ΔxylB genetic background exhibited similar reductions in xylanase and cellulase activity as ΔxylA but retaine higher necrotizing activity and virulence. This further supports the hypothesis that the enzymatic activity and/or necrotizing activity of xylA is not per se the key virulence determinant of *F. graminearum*, but rather it is the network‐level consequences of its disruption in the WT genetic background that cause the reduced virulence of the ΔxylA mutant, although the mechanisms responsible for these differences remain to be elucidated.

Finally, our extended characterization demonstrates that the *xylB* gene is not dispensable for host colonization, because its deletion led to significantly reduced fungal biomass and DON accumulation in wheat spikes despite the absence of a corresponding reduction in visible disease symptoms. This apparent discrepancy may reflect the fact that spikelet bleaching is not determined solely by fungal biomass but also by host physiological responses, including vascular occlusion and the consequent impairment of water transport through the rachis. Indeed, Fabre et al. (2019) did not find a clear correlation between fungal biomass, DON synthesis rate in spikes and severity of FHB symptoms in wheat.

In conclusion, taken together these results highlight the need to evaluate gene function in diverse genetic backgrounds and to account for possible compensatory mechanisms and interdependent traits when studying pathogenicity factors.

## 4 Experimental Procedures

### 4.1 Fungal Culture Conditions

*Fusarium graminearum* WT (PH1 strain) and mutant strains were cultured at 25°C on potato dextrose agar (PDA; Difco Laboratories). Conidia were obtained by growing the strains in a CMC (Sigma‐Aldrich)‐containing liquid medium, as reported in Sella et al. (2016).

### 4.2 Construction of the Gene Replacement Vector, Fungal Transformation‐Mediated Gene Disruption, Complementation and Mutants Screening

To knock‐out the *F. graminearum xylA* gene, a split‐marker construct carrying the geneticin resistance gene (*Gen*) was generated. The upstream and downstream flanking regions required for homologous recombination were amplified by PCR using *F. graminearum* genomic DNA as template. Specific primers (FG_10999UP_For/Rev for the upstream region, FG_10999DWN_For/Rev for the downstream region) (Figure S11a and Table S1) were used. Amplification was performed using REDTaq ReadyMix PCR Reaction Mix (Sigma‐Aldrich) in a final volume of 50 μL under the following conditions: 94°C for 4 min; then 35 cycles of 94°C for 1 min, 55°C for 30 s, and 72°C for 1 min.

The PCR products were purified and used in a fusion PCR to assemble the flanking regions with the *Gen* cassette (Figure S11b). Fusion PCR was carried out in a 25 μL volume containing REDTaq ReadyMix, 150 ng of each purified flanking region product (with homologous tails matching the 5′ and 3′ ends of the *Gen* gene) and 450 ng of the *Gen* gene obtained from the pII99 vector (Quarantin et al. 2016). The reaction conditions were 94°C for 3 min; then 20 cycles of 94°C for 1 min, 60°C for 1 min, and 72°C for 5 min. The fusion PCR product was used as template in two nested PCRs with the primer pairs 10999Nested_For/Rev (Table S1 and Figure S11c) to generate the two split‐marker fragments. Nested PCR conditions were as follows: 94°C for 4 min, followed by 35 cycles of 94°C for 1 min, 55°C for 30 s, and 72°C for 3 min. The resulting amplicons were gel‐purified and cloned into the pGEM‐T Easy vector (Promega) following the manufacturer's protocol. Recombinant *E. coli* colonies were screened, and positive clones were grown in high‐salt Luria Bertani broth (Fluka, BioChemika). Plasmid DNA was extracted using the NucleoBond MIDI kit (Promega) according to the manufacturer's instructions.

The resulting constructs of 2980 and 2920 bp were excised from the pGEM‐T Easy vector by digestion with NotI (Promega) or ApaI and SacI (ThermoScientific) and used to transform protoplasts of both the *F. graminearum* WT and the previously described ΔxylB.11 mutant strain (Sella et al. 2013).

Geneticin‐resistant colonies were isolated and subcultured on CMA plates supplemented with 150 μg/mL geneticin (Sigma‐Aldrich). Resistant transformants were single‐conidiated and preliminarily screened by PCR using primers targeting internal regions of the *xylA* gene (FG_10999_int_F/R) and of the *Gen* resistance gene (Gen_For/Rev) (Table S1 and Figure S11a,b). PCR conditions were as follows: 94°C for 3 min, followed by 35 cycles of 94°C for 30 s, 55°C for 30 s and 72°C for 1 min. Correct integration of the disruption cassette at the *xylA* locus was further confirmed by PCR amplification, performed as reported above except for a 2 min extension step at 72°C of the external flanking regions of the disruption cassette with the Check_for/rev primers reported in Table S1 and Figure S12c.

To generate the ΔΔxylA+B double mutant, a cassette containing the hygromycin (*hyg*) resistance gene previously described by Sella et al. (2013) was used to transform protoplasts of the ΔxylA_3F single mutant strain obtained as described above. The resistant transformants were single‐conidiated and preliminarily screened by PCR (94°C for 3 min, followed by 35 cycles of 94°C for 30 s, 55°C for 30 s and 72°C for 1 min) using primers targeting internal regions of the *xylB* gene (FG_03624_int_F/R) and of the *hyg* resistance gene (HygInt_For/Rev; Table S1 and Figure S12a,b). Correct integration of the disruption cassette at the *xylB* locus was further confirmed by amplifying the external flanking regions of the disruption cassette as above reported (Figure S12c), using an annealing temperature of 57°C.

To complement the ΔxylA mutant, protoplasts of the ΔxylA_3F strain were co‐transformed with the *xylA* gene (1113 bp of promoter region, 802 bp of open reading frame and 963 bp of terminator region) and the *hyg* resistance gene, amplified using the primer pairs reported in Table S1 at an annealing temperature of 56°C. Complemented mutants were screened with the primer pairs HygInt_For/Rev and XylAcompl‐F/R and grown on PDA or PDA supplemented with 100 μg/mL geneticin.

Copy number of *Gen*, *hyg* or *xylA* genes in the mutants' genome was quantified by qPCR following the protocol described by Solomon et al. (2008). Genomic DNA was extracted from 100 mg of fungal mycelium with the DNeasy Plant Mini Kit (Qiagen) and used as template in a qPCR performed using a Rotor‐Gene Q thermocycler (Qiagen). Reactions were carried out with the iQ SYBR Green Supermix (Bio‐Rad) and primer pairs specific for *Gen* (qGen_for/rev), *hyg* (qHyg_for/rev) or *xylA* (10999RTfor/rev) genes (Table S1). The *F. graminearum* translation elongation factor 1‐α gene (*TEF1*; *FGSG_08811*), present in a single copy in the fungal genome, was used as reference. Thermal cycling conditions were as follows: initial denaturation at 95°C for 2 min, followed by 40 cycles at 95°C for 5 s and 60°C for 10 s. Data analysis was performed using the Rotor‐Gene v. 2.0.3.2 software.

### 4.3 Growth Assays and Quantification of Enzymatic Activity

Radial growth of *F. graminearum* WT and mutant strains was evaluated on CMA plates. Conidia (1000) of WT and mutant strains were spotted onto CMA plates and incubated at 25°C for 7 days to measure colony diameters.

To evaluate total xylanase, cellulase, polygalacturonase activities and necrotizing activity, 1 × 10^4^ conidia mL^−1^ of WT and mutant strains were inoculated into 20 mL of Szécsi minimal liquid medium (Szecsi 1990) supplemented with one of the following carbon sources: 0.5% (w/v) beechwood xylan (Megazyme, Neogen), 0.5% (w/v) medium‐viscosity CMC, 0.5% (w/v) apple pectin (esterification 70%–75%, Sigma‐Aldrich), or 2 g of wheat spikelets ( *T. aestivum* ‘Bobwhite’), collected at flowering stage, to evaluate necrotizing activity. Cultures were incubated at 25°C on an orbital shaker at 110 rpm for 4 days.

After incubation, mycelia were harvested by filtration through sterile Miracloth, washed twice with deionized water, dried at 80°C for 3 days, and weighed. Culture supernatants were filtered through a 0.2 μm membrane and used for enzymatic or necrotizing activity assays.

Total xylanase activity was quantified using the dinitrosalicylic acid (DNSA) assay described by Sella et al. (2013). Briefly, 50 μL of culture supernatant was added to a 200 μL reaction mixture containing 0.5% beechwood xylan (w/v). d‐xylose (Merck) was used to generate a standard calibration curve. One unit of xylanase activity was defined as the amount of enzyme required to release 1 μmol of xylose under the assay conditions.

Cellulase and polygalacturonase activities were determined viscometrically, following the methods described respectively by Quarantin et al. (2019) and Tomassini et al. (2009). For each assay, 500 μL of culture filtrate was incubated with 0.25% (w/v) medium‐viscosity CMC (for cellulase) or 0.25% (w/v) polygalacturonic acid sodium salt (PGA; Sigma‐Aldrich) for 40 min. One unit of enzymatic activity was defined as the amount of enzyme that caused a 10% reduction in the initial relative viscosity of the substrate.

### 4.4 Fungal Virulence Assays on Soybean Seedling and Wheat Spikes

Soybean ( *Glycine max* ‘P15A20’) seedling infection assay was performed following the rolled‐towel method described by Sella et al. (2014). Each experiment was performed by inoculating 20 seeds per fungal strain. At 7 dpi, disease severity index was calculated as the ratio between lesion length on both root and hypocotyl and total seedling length, expressed as a percentage. An ectopic mutant strain, referred to as ΔΔECT and corresponding to a ΔxylB mutant carrying an ectopic insertion of the geneticin resistance gene, was also included in this virulence assay.

Wheat plants ( *T. aestivum* ‘Bobwhite’) were grown and inoculated at anthesis as reported in Sella et al. (2016). Each experiment was performed by inoculating at least seven spikes per fungal strain. At 21 dpi, spikes were collected for disease evaluation, fungal biomass quantification, and DON mycotoxin quantification. Disease severity was assessed as the percentage of symptomatic spikelets over the total number of spikelets per spike.

### 4.5 Fungal Biomass and DON Quantification in Infected Spikes

Wheat spikes infected and collected as above reported were frozen in liquid nitrogen and ground into a fine powder using the MM400 TissueLyser vibration mill (Retsch). The resulting homogenized tissue was used for fungal biomass and DON quantification.

Fungal biomass was quantified by qPCR following the protocol described by Moscetti et al. (2013). Total DNA was extracted from 100 mg of spike homogenized tissue using the DNeasy Plant Mini Kit (Qiagen), according to the manufacturer's instructions. Total DNA concentration was adjusted to 50 ng/μL for each sample and 5 ng was used as template in a qPCR performed using the QuantiNova SYBR Green PCR Kit (Qiagen) with primer pairs specific for the *TRI6* gene (Tri6_10F and Tri6_4R; Horevaj et al. 2011; Table S1). The *T. aestivum actin* gene (*AB181991*) was used as a reference using primers TaActF and TaActR (Table S1). Reactions were carried out on a Rotor‐Gene Q thermocycler (Qiagen). The thermal cycling conditions were as follows: initial denaturation at 95°C for 3 min, followed by 40 cycles at 95°C for 5 s and 60°C for 10 s. Data were analysed using the Rotor‐Gene v. 2.0.3.2 software (Qiagen) and the REST tool (Relative Expression Software Tool), employing the 2^−ΔΔ*C*t^ method (Pfaffl et al. 2002). Fungal biomass of mutants was calculated by setting that of the WT strain as 1.

Quantification of DON was carried out using the Bio‐Shield Deoxynivalenol ME kit (Prognosis Biotech), following the manufacturer's instructions. Data were analysed using Prognosis‐Data‐Reader software (Prognosis Biotech).

### 4.6 Gene Expression Analysis

Expression analysis during wheat spike infection of *F. graminearum* endoxylanase genes and other pathogenicity‐related genes was performed. *T. aestivum* ‘Bobwhite’ spikes were point‐inoculated as previously described. Infected spikelets were collected at 3 dpi, immediately frozen in liquid nitrogen, and ground to a fine powder using TissueLyser (Qiagen). The xylA_5C mutant was not included in this analysis, as it was found to lack an additional portion of the *xylA* locus beyond the region deleted by the insertion of the geneticin resistance cassette (Figure S1c).

Total RNA was extracted from 50 mg of powdered tissue using the Total RNA Purification Kit (Norgen Biotek Corp.), following the manufacturer's instructions. cDNA was synthesized from 1 μg of total RNA using the ImProm‐II Reverse Transcription System (Promega). The resulting cDNA was diluted 1:10 in RNase‐free water. qPCR was performed using the Rotor‐Gene Q thermocycler (Qiagen) and the iQ SYBR Green Supermix (Bio‐Rad). Gene expression analysis was performed on the *F. graminearum* genes encoding (i) the endoxylanases *xylB* (*FGSG_03624*), *xylA* (*FGSG_10999*), *FGSG_11487, FGSG_06445, FGSG_11304*, and *FGSG_11258*; (ii) the secreted effector proteins *FGSG_01831, FGSG_03600, FGSG_10212*, the latter encoding a cerato‐platanin; (iii) the enzymes *FGSG_08079* and *FGSG_02326*, involved in the biosynthesis of butenolide and aurofusarin, respectively; and (iv) the glucan 1,3‐β‐glucosidase *FGSG_06616*. The *F. graminearum* β‐tubulin gene (*FGSG_06611*) was used as a reference gene. The qPCR protocol consisted of an initial step at 95°C for 3 min, followed by 40 cycles of 95°C for 5 s and 60°C for 15 s. The list of primers used for the expression analysis is provided in Table S1. For expression analysis of *F. graminearum* endoxylanase genes, primers from Sella et al. (2013) were used.

Data were analysed using the Rotor‐Gene v. 2.0.3.2 software (Qiagen) and the REST tool employing the 2^−ΔΔ*C*t^ method.

For *xylA* gene expression in the complemented strains, total RNA was extracted from 100 mg of fungal mycelia grown for 4 days in CMC liquid medium. cDNA synthesis and qPCR were performed as reported above. Data were analysed using the Rotor‐Gene v. 2.0.3.2 software (Qiagen). Primers used are reported in Table S1.

### 4.7 Quantification of Necrotizing Activity

To assess necrotizing activity, 50 μL of culture filtrates from each strain, grown on a medium containing spikelets of *T. aestivum* ‘Bobwhite’ collected at flowering stage, were infiltrated into at least three fully expanded leaves (four infiltration sites per leaf) of 2‐month‐old *N. benthamiana* plants (cv. SR1). Leaf images were acquired and necrotic lesion areas were quantified at 4 days post‐infiltration using ImageJ2 software. Total protein concentration in culture filtrates was determined using the Bradford assay (Sigma‐Aldrich) according to the manufacturer's instructions. Briefly, 30 μL of each culture filtrate diluted 1:10 was used for protein quantification and three independent biological replicates were analysed. DON levels in the culture filtrates were also quantified as above reported.

### 4.8 Statistical Analysis

Fungal biomass quantification in infected spikes was analysed using Dunnett's multiple comparisons test to compare each mutant to the WT strain. All other datasets were evaluated through one‐way ANOVA followed by Tukey's post hoc test for multiple comparisons. Statistical analyses were performed using the RStudio software (R v. 2024.12.1+563; RStudio Team).

## Author Contributions

**Arianna Panichi:** methodology, investigation, formal analysis, validation, visualization, writing – original draft. **Silvana Odorizzi:** investigation, visualization. **Carla Castiglioni:** investigation. **Silvio Tundo:** validation, writing – review and editing. **Alessandro Raiola:** validation, writing – review and editing. **Francesco Favaron:** validation, writing – review and editing. **Wilhelm Schäfer:** validation, writing – review and editing. **Luca Sella:** conceptualization, supervision, validation, visualization, writing – review and editing, resources, funding acquisition, project administration.

## Conflicts of Interest

The authors declare no conflicts of interest.

## Supporting information


**Figure S1:** PCR screening of ΔxylA single mutant strains obtained using wild‐type (WT) PH1 strain as genetic background and of ΔΔxylB+A double mutant obtained using ΔxylB as genetic background. Primers internal to the *xylA* gene (a) and to the geneticin resistance gene (b) were used to screen putative mutants. Three knock‐out (KO) mutants for the *xylA* gene, named 1F, 3F and 5C (lanes 2, 3 and 4, respectively), and one double KO mutant, named ΔΔxylB+A (lane 5), were analysed. Amplification of the upstream (c) and downstream (d) regions flanking the cassette used to obtain the KO mutants confirms homologous recombination in the three single mutant strains and in the ΔΔxylB+A double mutant. (a, b) Lane 1: *Fusarium graminearum* wild‐type (PH1 strain), used as control; lane 2: ECT_1A; lane 3: ECT_2C; lane 4: ΔxylA_5C; lane 5: ECT_2D; lane 6: ΔxylA_1F; lane 7: ΔxylA_3F; lane 8: ΔΔxylB+A double KO mutant; lane 9: no template control. (c, d) Lane 1: *F. graminearum* WT; lane 2: ΔxylA_1F; lane 3: ΔxylA_3F; lane 4: ΔxylA_5C; lane 5: ΔΔxylB+A double KO mutant. M = marker 1Kb + (ThermoScientific). Only the ΔxylA_5C single mutant (c; lane 4) failed to amplify the upstream region of the integration locus.

**Figure S2:** Quantification of geneticin (for ΔxylA_1F, ΔxylA_3F, ΔxylA_5C and ΔΔxylB+A) and hygromycin (for ΔΔxylA+B) copy number in the mutants' genome. Quantification was carried out by quantitative PCR following the protocol described by Solomon et al. (2008) and using the *Fusarium graminearum* translation elongation factor 1‐α (*TEF1*), present in a single copy in the fungal genome, as a reference gene. Relative expression levels were analysed using the Rotor‐Gene v. 2.0.3.2 software (Qiagen). Data represent the mean ± standard deviation of three replicates.

**Figure S3:** Vegetative growth of *Fusarium graminearum* wild‐type (WT) and xylanase mutant strains on complete medium agar (CMA). (a) Radial growth was measured after 7 days at 25°C and expressed in cm^2^ as the average of at least three independent replicates per strain. Bars indicate standard errors. Statistical analyses were conducted using one‐way ANOVA followed by Tukey–Kramer post hoc test. Different letters indicate significant differences at *p* < 0.05. (b) Mycelia growth in CMA plates photographed at 7 days post‐inoculation.

**Figure S4:** Mycelia dry weight produced by wild‐type (WT) and mutant strains in xylanase (a), cellulase (b) and polygalacturonase (c) inducing media. Conidia (1 × 10^4^ mL^−1^) were grown in xylan‐, carboxymethyl cellulose (CMC)‐ or pectin‐containing Szécsi liquid media for 4 days. Mycelia were then filtered, washed twice with sterile water, and dried at 80°C for 4 days. Data are presented as the average of three independent experiments for each inducing medium. Bars indicate standard deviation. Statistical analyses were conducted using one‐way ANOVA followed by Tukey–Kramer post hoc test. Different letters indicate significant differences at *p* < 0.05.

**Figure S5:** (a) Representative pictures of necrotic areas in *Nicotiana benthamiana* leaves infiltrated with fungal culture filtrates obtained growing conidia of *Fusarium graminearum* wild‐type (WT) and mutant strains in Szécsi liquid medium supplemented with wheat spikelets at the flowering stage. One hundred microlitres of culture filtrate were infiltrated into *N. benthamiana* ‘SR1’ leaves. A negative control (CT−), consisting of Szécsi medium supplemented with wheat spikelets, was included. (b) Quantification of proteins secreted by *F. graminearum* WT and mutant strains in culture filtrates after 4 days of growth in Szécsi liquid medium supplemented with wheat spikelets. Total protein concentration (μg/mL) was determined using the Bradford assay subtracting from all samples the protein concentration measured in the uninoculated medium. Bars represent the mean ± SE of three independent biological replicates. Statistical analyses were conducted using one‐way ANOVA followed by Tukey–Kramer post hoc test. Different letters indicate significant differences at *p* < 0.05.

**Figure S6:** Summary phenotypes displayed by ΔxylA, ΔxylB and ΔΔxylB+A mutants compared to *Fusarium graminearum* wild‐type (PH1 strain). The ΔΔxylB+A mutant was obtained by disrupting the *xylA* gene in the ΔxylB genetic background.

**Figure S7:** PCR screening of double knock‐out (KO) mutant obtained using the ΔxylA_3F strain as genetic background. Primers internal to the *xylB* gene (a) and to the hygromycin resistance gene (b) were used to screen putative mutants. Three ectopic mutants (a, b; lanes 1, 2 and 4) and one double KO mutant, named ΔΔxylA+B (a, b; lane 3), were obtained. Amplification of the UP (c) and DOWN (d) regions flanking the cassette used to obtain the KO mutants confirms homologous recombination in the ΔΔxylA+B double KO mutant. (a, b) Lanes 1, 2 and 4: ectopic mutants; lane 3: ΔΔxylA+B double KO mutant; lane 5: *Fusarium graminearum* wild‐type (WT; PH1strain). (c, d) Lane 1: WT (PH1 strain); lane 2: ΔΔxylA+B mutant strain; M = marker 1 kb + (ThermoScientific).

**Figure S8:** (a, b) PCR screening of the complemented strains obtained from the ΔxylA_3F mutant. Primers amplifying the *Fusarium graminearum xylA* full open reading frame (a) and the hygromycin (*hyg*) resistance gene (b) were used to screen putative complemented strains. Lane 1: C2.1; lane 2: C3.1; lane 3: C3.8; lane 4: C4.3; lane 5: C4.4; lane 6: C4.5; lane 7: C4.6; lane 8: no template control. M = marker 1 kb + (ThermoScientific). (c) Growth of C2.1 and C3.8 in potato dextrose agar (PDA) supplemented or not with 100 μg/mL geneticin (GEN). 1: C2.1 in PDA + GEN; 2: C3.8 in PDA + GEN; 3: C2.1 in PDA; 4: C3.8 in PDA. (d) Quantification of *xylA* and *hyg* copy number in the genome of the complemented strains. Quantification was carried out by quantitative PCR (qPCR) following the protocol described by Solomon et al. (2008) and using the *F. graminearum* translation elongation factor 1‐α (*TEF1*), present in a single copy in the fungal genome, as a reference gene. Relative expression levels were analysed using the Rotor‐Gene v. 2.0.3.2 software (Qiagen). Data represent the mean ± standard deviation of three replicates. (e) Expression analysis of *F. graminearum xylA* gene. Total RNA was extracted from mycelia of wild‐type (WT), C2.1 and C3.8 complemented strains collected after 4 days of growth in carboxymethyl cellulose (CMC) liquid medium. Reverse transcription‐qPCR was performed using the *F. graminearum β‐tubulin* gene as housekeeping. Relative expression levels were analysed using the Rotor‐Gene v. 2.0.3.2 software (Qiagen). Data represent the mean of at least three replicates. Error bars indicate standard deviation.

**Figure S9:** (a, b) Vegetative growth of *Fusarium graminearum* wild‐type (WT), ΔxylA_3F mutant strain and C2.1 and C3.8 complemented strains on complete medium agar (CMA). (a) Radial growth was measured after 7 days at 25°C and expressed in cm^2^ as the average of at least three independent replicates per strain. Bars indicate standard deviation. Statistical analyses were conducted using one‐way ANOVA followed by Tukey–Kramer post hoc test. Different letters indicate significant differences at *p* < 0.05. (b) Mycelial growth in CMA plates photographed at 7 days post‐inoculation. (c) Total xylanase activity secreted by *F. graminearum* WT, ΔxylA_3F, C2.1 and C3.8 strains after 4 days of growth in Szécsi liquid medium supplemented with beechwood xylan. The enzymatic activity was measured using the DNSA method, and one xylanase unit was defined as the amount of enzyme required to release 1 μmol of xylose under the assay conditions. Data are presented as mean ± standard error of four independent replicates. Statistical analyses were conducted using one‐way ANOVA followed by Tukey–Kramer post hoc test. Different letters indicate significant differences at *p* < 0.05.

**Figure S10:** Virulence assays of complemented strains on wheat spikes. (a) *Triticum aestivum* ‘Bobwhite’ spikes were point inoculated at anthesis with 2000 conidia, and disease symptoms were evaluated at 21 days post‐inoculation (dpi) by counting the percentage of infected spikelets. Data are presented as mean ± standard error of at least three independent experiments, each performed inoculating at least five wheat spikes per fungal strain. Statistical analyses were conducted using one‐way ANOVA followed by Tukey–Kramer post hoc test. Different letters indicate significant differences at *p* < 0.05. Infected spikes were collected at 21 dpi to quantify (b) fungal biomass by quantitative PCR and (c) deoxynivalenol (DON) contamination by ELISA test. Data are presented as average values of at least three independent experiments. Bars represent standard errors. Dunnett's multiple comparisons test was applied for fungal biomass analysis. ANOVA followed Tukey–Kramer post hoc test was used for DON quantification. Different letters indicate significant differences at *p* < 0.05. WT, wild‐type strain.

**Figure S11:** Split marker disruption cassette containing the geneticin resistance gene (*Gen*) used for replacing the *Fusarium graminearum FGSG_10999* (*xylA*) gene in both the wild‐type (PH1 strain) and the ΔxylB mutant strain. (a) Amplification of the flanking homology regions. (b) Fusion of the homologous flanking regions to the antibiotic resistance marker gene. (c) Amplification of the split marker constructs 10999_SPLIT1_Gen (5′) and 10999_SPLIT2_Gen (3′) using nested primers.

**Figure S12:** First PCR screening with forward and reverse primers (black arrows) designed to amplify (a) the internal regions of the geneticin or hygromycin resistance genes (primers 1 and 2) or (b) the *Fusarium graminearum xylA* or *xylB* gene (primers 3 and 4). (c) Second PCR screening with forward and reverse primers (black arrows) designed to amplify the upstream (primers 3 and 4) and downstream (primers 5 and 6) regions flanking the cassette used to obtain the knock‐out mutants.

**Table S1:** Primers used in the present study.

## Data Availability

Data are available from the corresponding author upon reasonable request.
