# Supplementary material for: Deletion of Major Endoxylanase Genes in Fusarium graminearum Uncovers Distinct Virulence Phenotypes Influenced by the Genetic Background
Source: Mol Plant Pathol. 2026 Oct 27;27(10):e70362. doi: 10.1111/mpp.70362 (PMC13714102; doi:10.1111/mpp.70362)
Supplement: Supplementary file 1 [file MPP-27-e70362-s001.pdf]

FIGURE S9

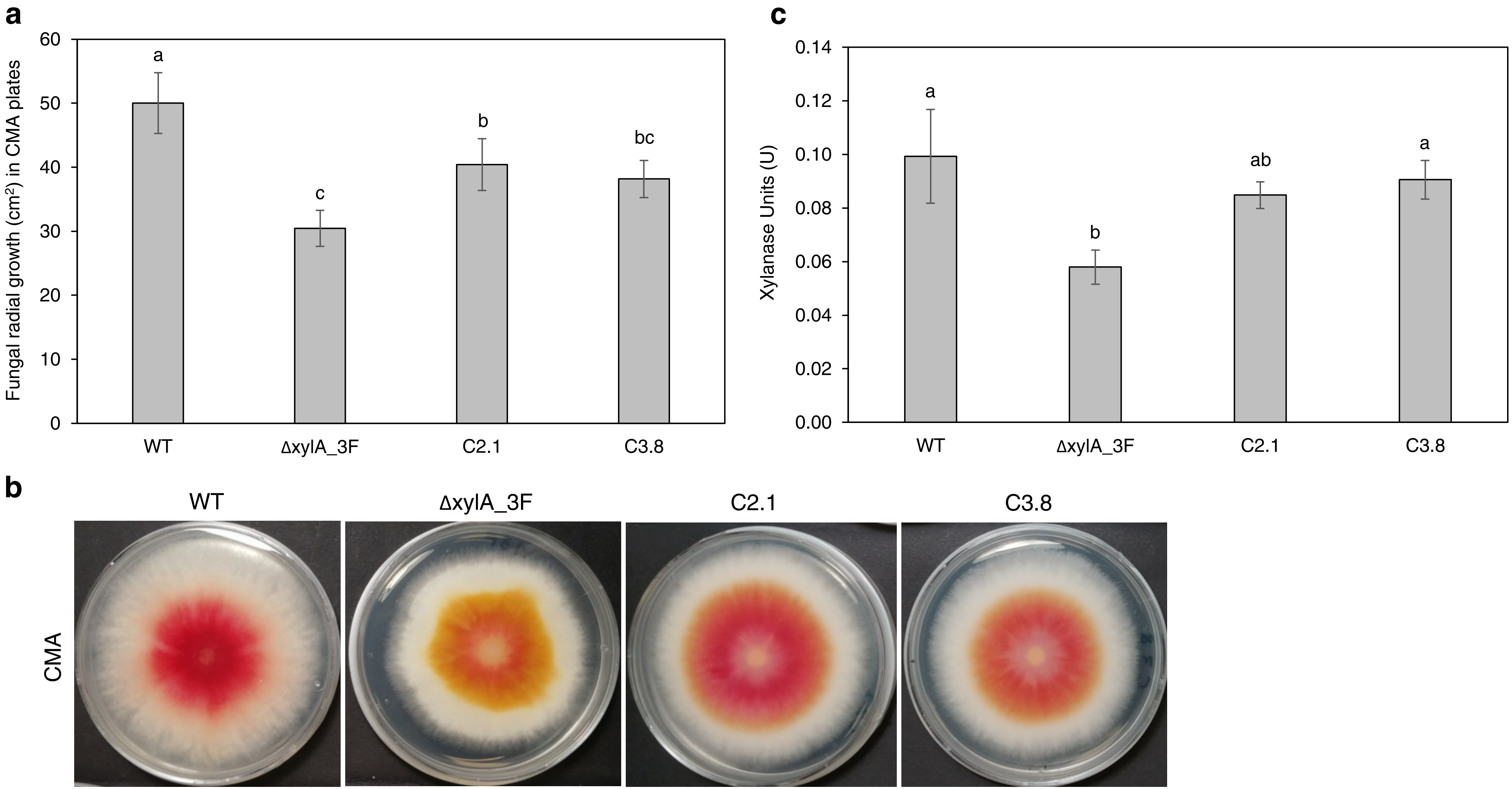

Figure S9. Vegetative growth of *F. graminearum* wild-type (WT), ΔxylA_3F mutant strain and C2.1 and C3.8 complemented strains on complete medium agar (CMA). (a) Radial growth was measured after 7 days at 25 °C and expressed in cm^2 as the average of at least three independent replicates per strain. Bars indicate standard deviation. Statistical analyses were conducted using one-way ANOVA followed by Tukey-Kramer post hoc test. Different letters indicate significant differences at $p < 0.05$. (b) Mycelia growth in CMA plates photographed at 7 dpi. (c) Total xylanase activity secreted by *Fusarium graminearum* WT, ΔxylA_3F, C2.1 and C3.8 strains after 4 days of growth in Szécsi liquid medium supplemented with beechwood xylan. Total xylanase activity was measured using the DNSA method, and one xylanase unit was defined as the amount of enzyme required to release one µmol of xylose under the assay conditions. Data are presented as mean ± standard error of four independent replicates. Statistical analyses were conducted using one-way ANOVA followed by Tukey-Kramer post hoc test. Different letters indicate significant differences at $p < 0.05$.
