# Supplementary material for: Deletion of Major Endoxylanase Genes in Fusarium graminearum Uncovers Distinct Virulence Phenotypes Influenced by the Genetic Background
Source: Mol Plant Pathol. 2026 Oct 27;27(10):e70362. doi: 10.1111/mpp.70362 (PMC13714102; doi:10.1111/mpp.70362)
Supplement: Supplementary file 2 [file MPP-27-e70362-s002.pdf]

FIGURE S1

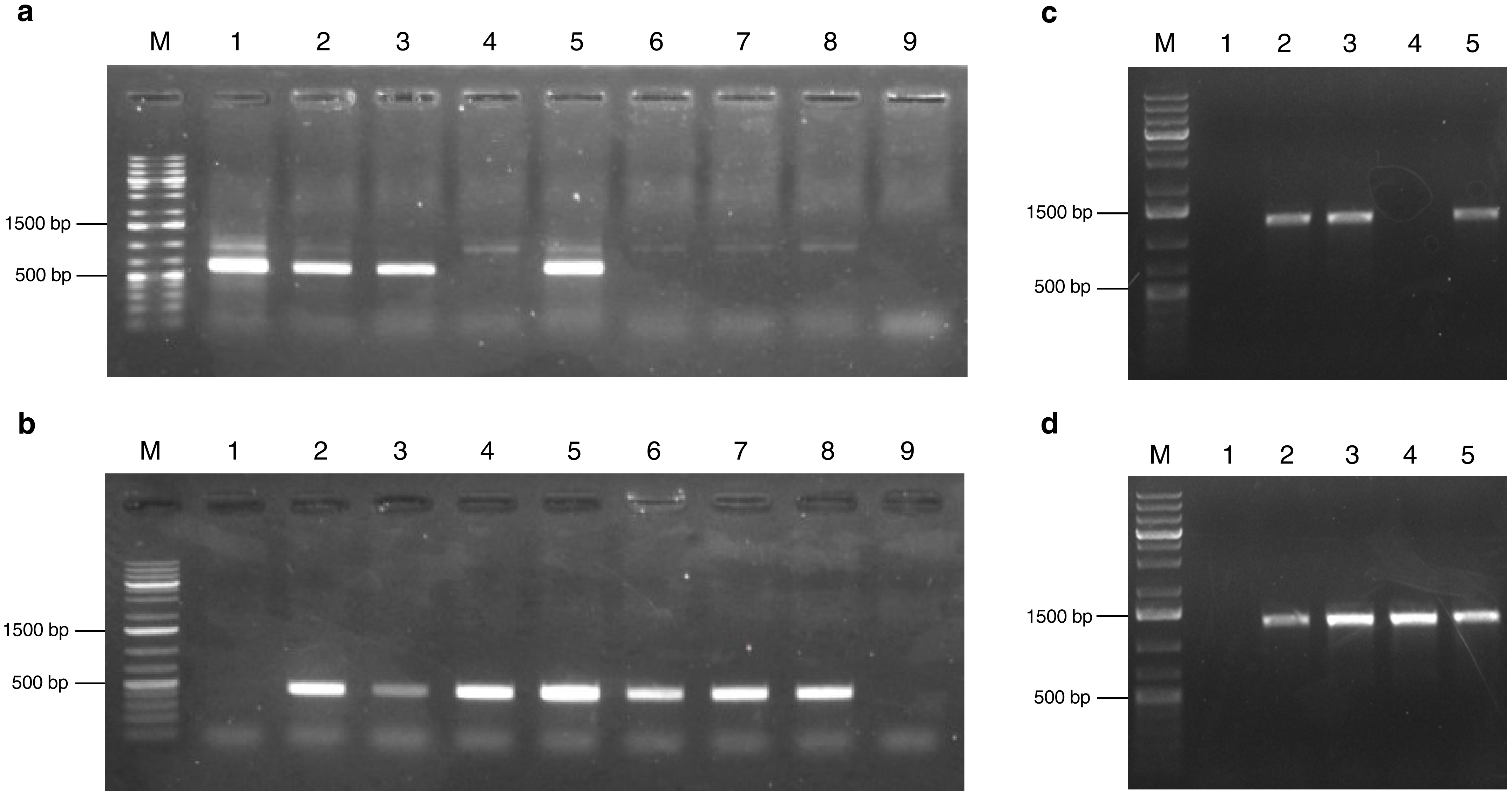

Figure S1. PCR screening of ΔxylA single mutant strains obtained using WT PH1 strain as genetic background and of ΔΔxylB+A double mutant obtained using ΔxylB as genetic background. Primers internal to the *xylA* gene (a) and to the geneticin resistance gene (b) were used to screen putative mutants. Three knock-out (KO) mutants for the *xylA* gene, named 1F, 3F and 5C (lanes 2, 3 and 4, respectively), and one double KO mutant, named ΔΔxylB+A (lane 5), were analysed. Amplification of the upstream (c) and downstream (d) regions flanking the cassette used to obtain the KO mutants confirms homologous recombination in the three single mutant strains and in the ΔΔxylB+A double mutant. (a-b) Lane 1: *F. graminearum* wild-type (PH1 strain), used as control; lane 2: ECT_1A; lane 3: ECT_2C; lane 4: ΔxylA_5C; lane 5: ECT_2D; lane 6: ΔxylA_1F; lane 7: ΔxylA_3F; lane 8: ΔΔxylB+A double KO mutant; lane 9: no template control. (c-d) Lane 1: *F. graminearum* WT; lane 2: ΔxylA_1F; lane 3: ΔxylA_3F; lane 4: ΔxylA_5C; lane 5: ΔΔxylB+A double KO mutant. M = marker 1Kb+ (ThermoScientific). Only the ΔxylA_5C single mutant (c, lane 4) failed to amplify the upstream region of the integration locus.
