# Supplementary material for: Deletion of Major Endoxylanase Genes in Fusarium graminearum Uncovers Distinct Virulence Phenotypes Influenced by the Genetic Background
Source: Mol Plant Pathol. 2026 Oct 27;27(10):e70362. doi: 10.1111/mpp.70362 (PMC13714102; doi:10.1111/mpp.70362)
Supplement: Supplementary file 3 [file MPP-27-e70362-s003.pdf]

FIGURE S12

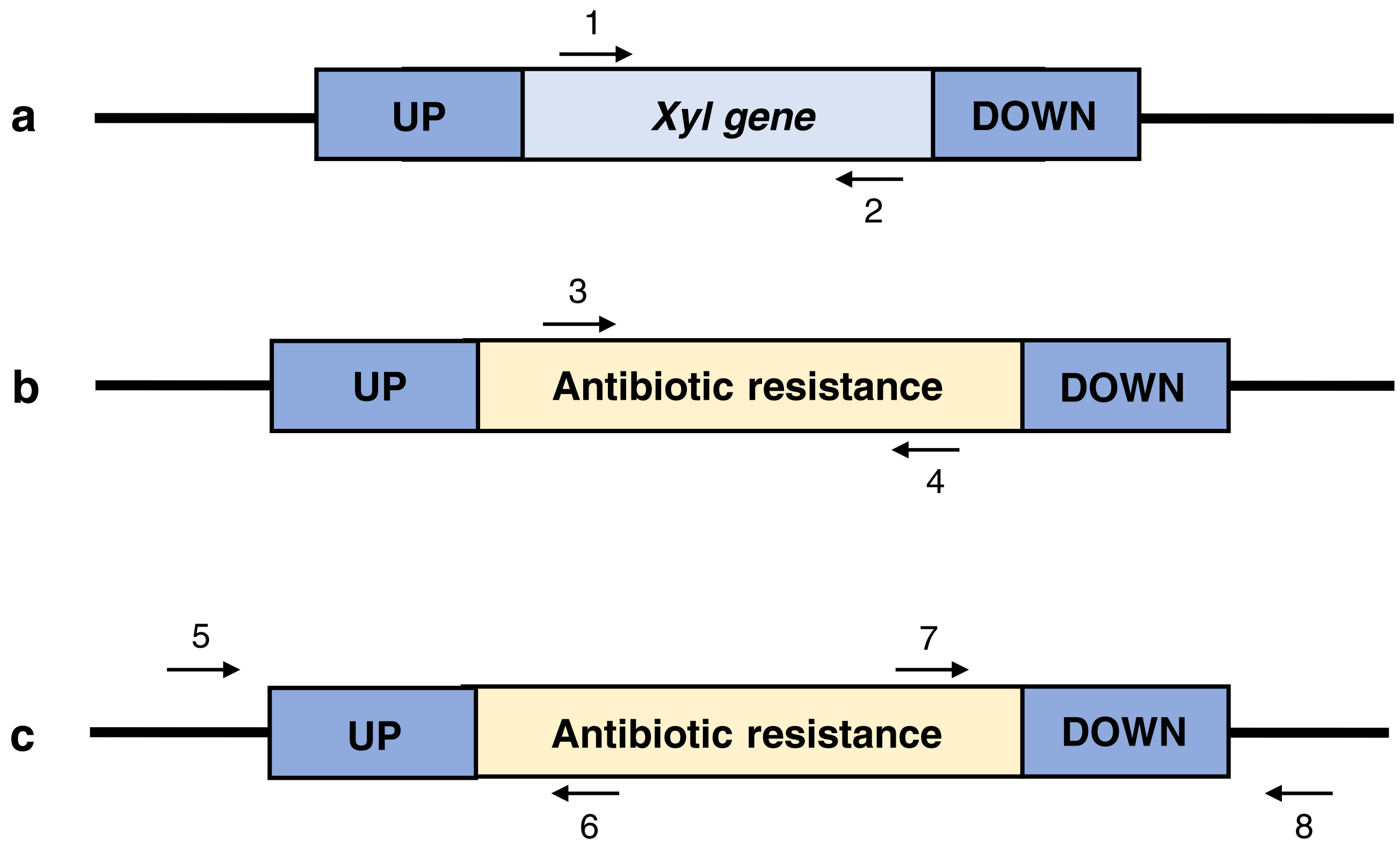

Figure S12. First PCR screening with forward and reverse primers (black arrows) designed to amplify (a) the internal regions of the geneticin or hygromycin resistance genes (primers 1 and 2) or (b) the *F. graminearum xylA* or *xylB* gene (primers 3 and 4). (c) Second PCR screening with forward and reverse primers (black arrows) designed to amplify the upstream (primers 3 and 4) and downstream (primers 5 and 6) regions flanking the cassette used to obtain the knock-out mutants.
