# Supplementary material for: Deletion of Major Endoxylanase Genes in Fusarium graminearum Uncovers Distinct Virulence Phenotypes Influenced by the Genetic Background
Source: Mol Plant Pathol. 2026 Oct 27;27(10):e70362. doi: 10.1111/mpp.70362 (PMC13714102; doi:10.1111/mpp.70362)
Supplement: Supplementary file 4 [file MPP-27-e70362-s004.pdf]

FIGURE S10

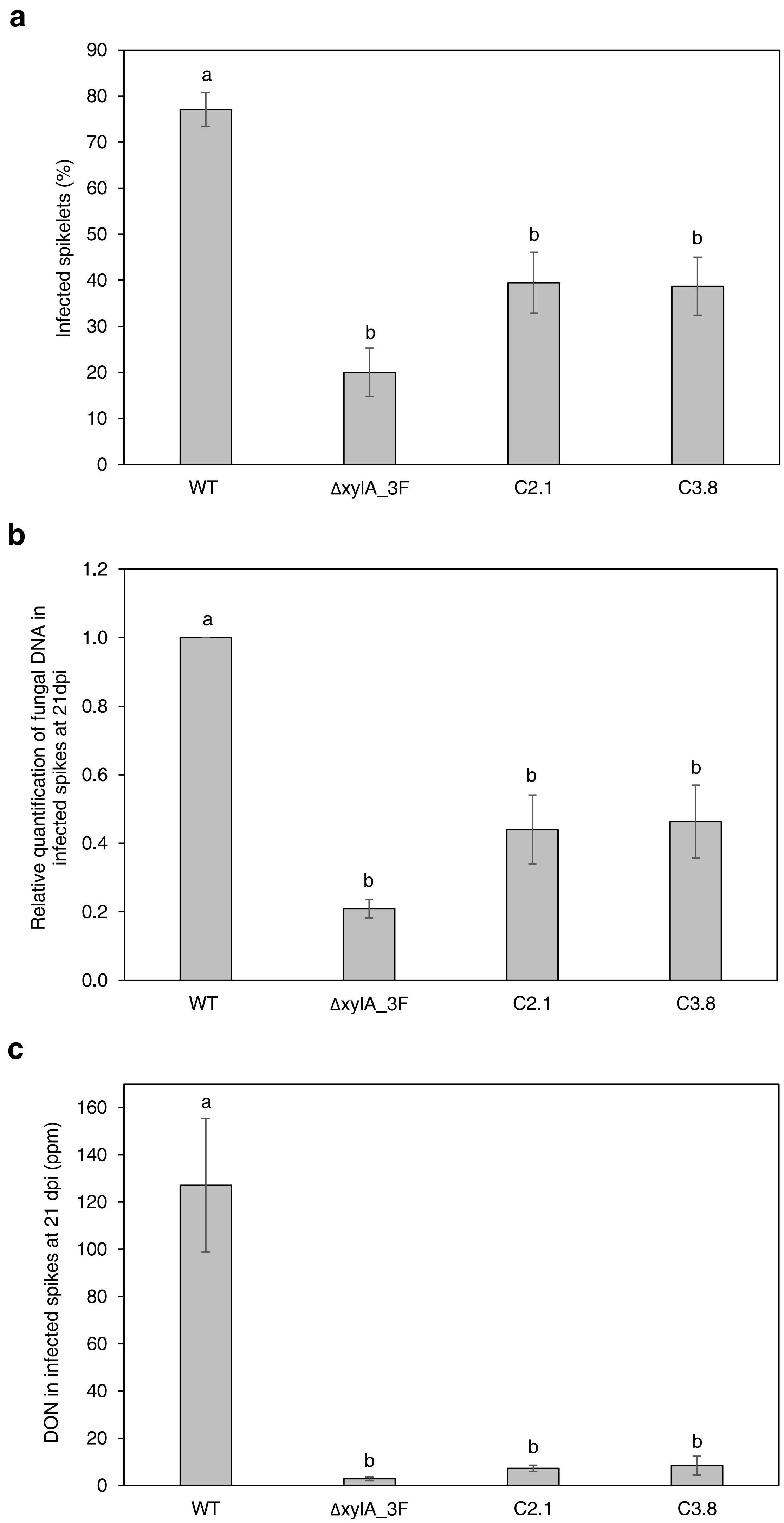

Figure S10. Virulence assays of complemented strains on wheat spikes. (a) *Triticum aestivum* cv. Bobwhite spikes were point inoculated at anthesis with 2,000 conidia, and disease symptoms were evaluated at 21 days post-inoculation (dpi) by counting the percentage of infected spikelets. Data are presented as mean ± standard error of at least three independent experiments, each performed inoculating at least five wheat spikes per fungal strain. Statistical analyses were conducted using one-way ANOVA followed by Tukey-Kramer post hoc test. Different letters indicate significant differences at $p < 0.05$. Infected spikes were collected at 21 dpi to quantify (b) fungal biomass by qPCR and (c) deoxynivalenol (DON) contamination by ELISA test. Data are presented as average values of at least three independent experiments. Bars represent standard errors. Dunnett's multiple comparisons test was applied for fungal biomass analysis. ANOVA followed Tukey-Kramer post hoc test was used for DON quantification. Different letters indicate significant differences at $p < 0.05$. WT: wild-type strain.
