# Supplementary material for: Deletion of Major Endoxylanase Genes in Fusarium graminearum Uncovers Distinct Virulence Phenotypes Influenced by the Genetic Background
Source: Mol Plant Pathol. 2026 Oct 27;27(10):e70362. doi: 10.1111/mpp.70362 (PMC13714102; doi:10.1111/mpp.70362)
Supplement: Supplementary file 5 [file MPP-27-e70362-s005.pdf]

FIGURE S6

Phenotype	ΔxylA	ΔxylB	ΔΔxylB+A
**Virulence on wheat/soybean**	↓↓↓	=	↓
**Fungal biomass in wheat spikes**	↓↓↓	↓↓	↓
**DON in wheat spikes**	↓↓↓	↓↓	↓↓↓
**Enzymatic activities**	↓↓	=	↓↓
**Necrotizing activity**	↓↓	↓	↓
**Endo-xylanase gene expression**	↓↓	=	=
**Effector gene expression**	↓↓	↑	=

Legend ↑ higher = similar ↓ lower

Figure S6. Summary phenotypes displayed by ΔxylA, ΔxylB and ΔΔxylB+A mutants compared to *F. graminearum* wild-type (PH1 strain). The ΔΔxylB+A mutant was obtained by disrupting the *xylA* gene in the ΔxylB genetic background.
