# Supplementary material for: Deletion of Major Endoxylanase Genes in Fusarium graminearum Uncovers Distinct Virulence Phenotypes Influenced by the Genetic Background
Source: Mol Plant Pathol. 2026 Oct 27;27(10):e70362. doi: 10.1111/mpp.70362 (PMC13714102; doi:10.1111/mpp.70362)
Supplement: Supplementary file 6 [file MPP-27-e70362-s006.pdf]

FIGURE S5

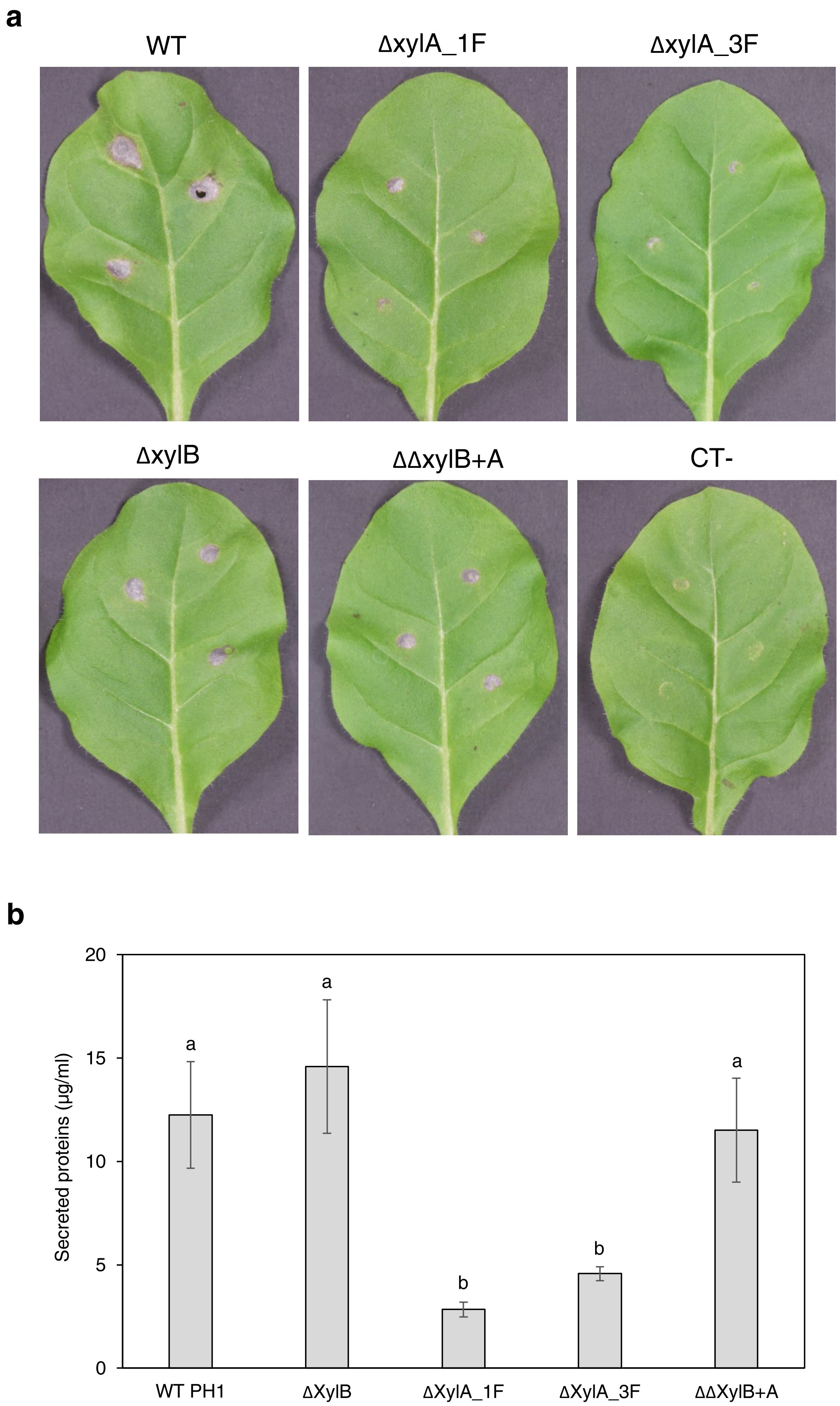

Figure S5. (a) Representative pictures of necrotic areas in tobacco leaves infiltrated with fungal culture filtrates obtained growing conidia of *Fusarium graminearum* wild-type (WT) and mutant strains in Szécsi liquid medium supplemented with wheat spikelets at the flowering stage. One hundred µL of culture filtrate were infiltrated into *Nicotiana benthamiana* cv. SR1 leaves. A negative control (CT−), consisting of Szécsi medium supplemented with wheat spikelets, was included. (b) Quantification of proteins secreted by *F. graminearum* WT and mutant strains in culture filtrates after 4 days of growth in Szécsi liquid medium supplemented with wheat spikelets. Total protein concentration (µg/ml) was determined using the Bradford assay subtracting from all samples the protein concentration measured in the uninoculated medium. Bars represent the mean ± SE of three independent biological replicates. Statistical analyses were conducted using one-way ANOVA followed by Tukey-Kramer post hoc test. Different letters indicate significant differences at $p < 0.05$.
