# Supplementary material for: Deletion of Major Endoxylanase Genes in Fusarium graminearum Uncovers Distinct Virulence Phenotypes Influenced by the Genetic Background
Source: Mol Plant Pathol. 2026 Oct 27;27(10):e70362. doi: 10.1111/mpp.70362 (PMC13714102; doi:10.1111/mpp.70362)
Supplement: Supplementary file 7 [file MPP-27-e70362-s007.pdf]

FIGURE S2

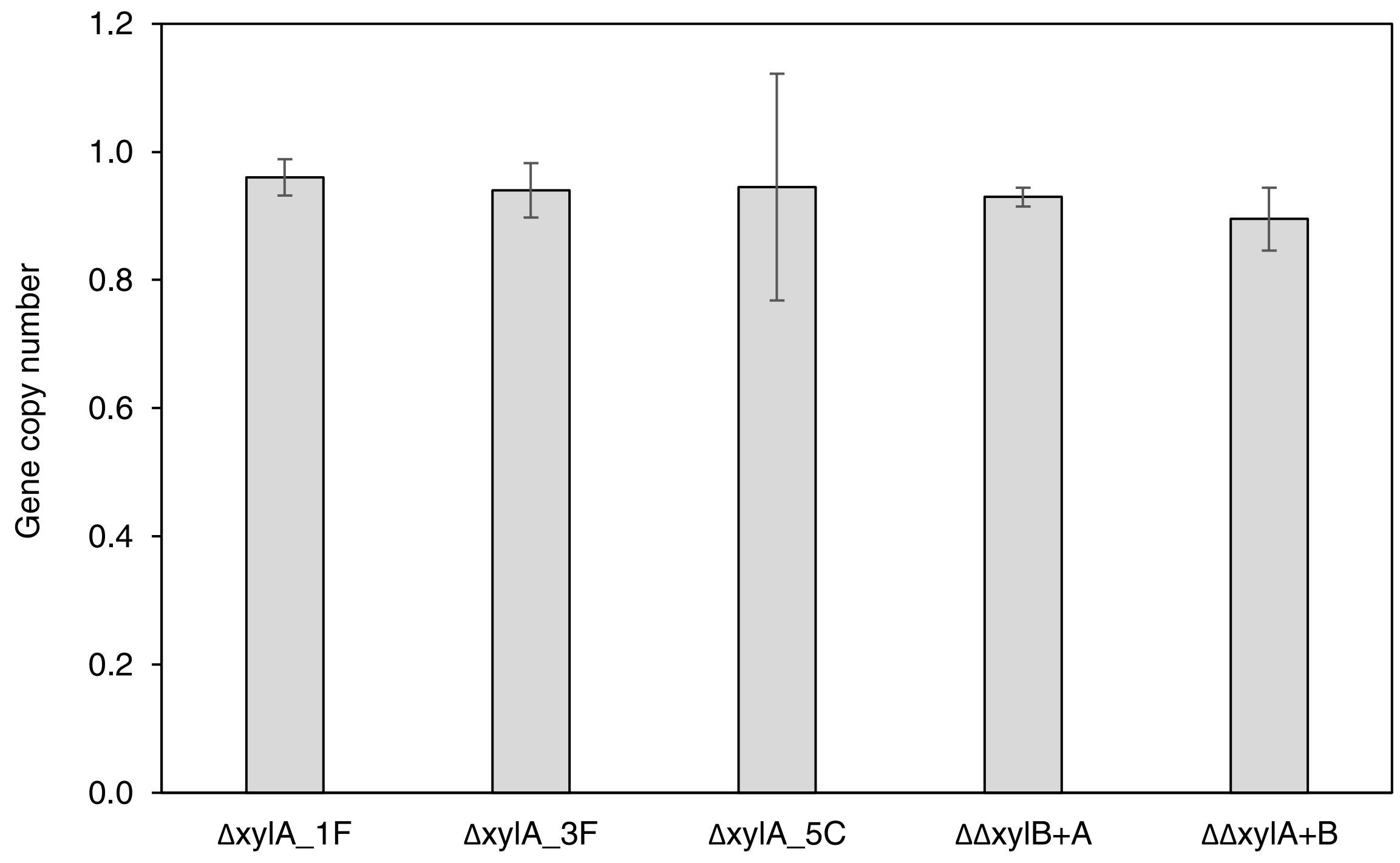

Figure S2. Quantification of geneticin (for ΔxylA_1F, ΔxylA_3F, ΔxylA_5C and ΔΔxylB+A) and hygromycin (for ΔΔxylA+B) copy number in the mutants' genome. Quantification was carried out by qPCR following the protocol described by Solomon et al. (2008) and using the *Fusarium graminearum* translation elongation factor 1-alpha (TEF1), present in a single copy in the fungal genome, as a reference gene. Relative expression levels were analyzed using the Rotor-Gene v. 2.0.3.2 software (Qiagen). Data represent the mean ± standard deviation of three replicates.

Solomon et al. (2008) A quantitative PCR approach to determine gene copy number. *Fungal Genetics Reports* 55: 5-8. https://doi.org/10.4148/1941-4765.1082.
