# Supplementary material for: Deletion of Major Endoxylanase Genes in Fusarium graminearum Uncovers Distinct Virulence Phenotypes Influenced by the Genetic Background
Source: Mol Plant Pathol. 2026 Oct 27;27(10):e70362. doi: 10.1111/mpp.70362 (PMC13714102; doi:10.1111/mpp.70362)
Supplement: Supplementary file 8 [file MPP-27-e70362-s008.pdf]

# FIGURE S11

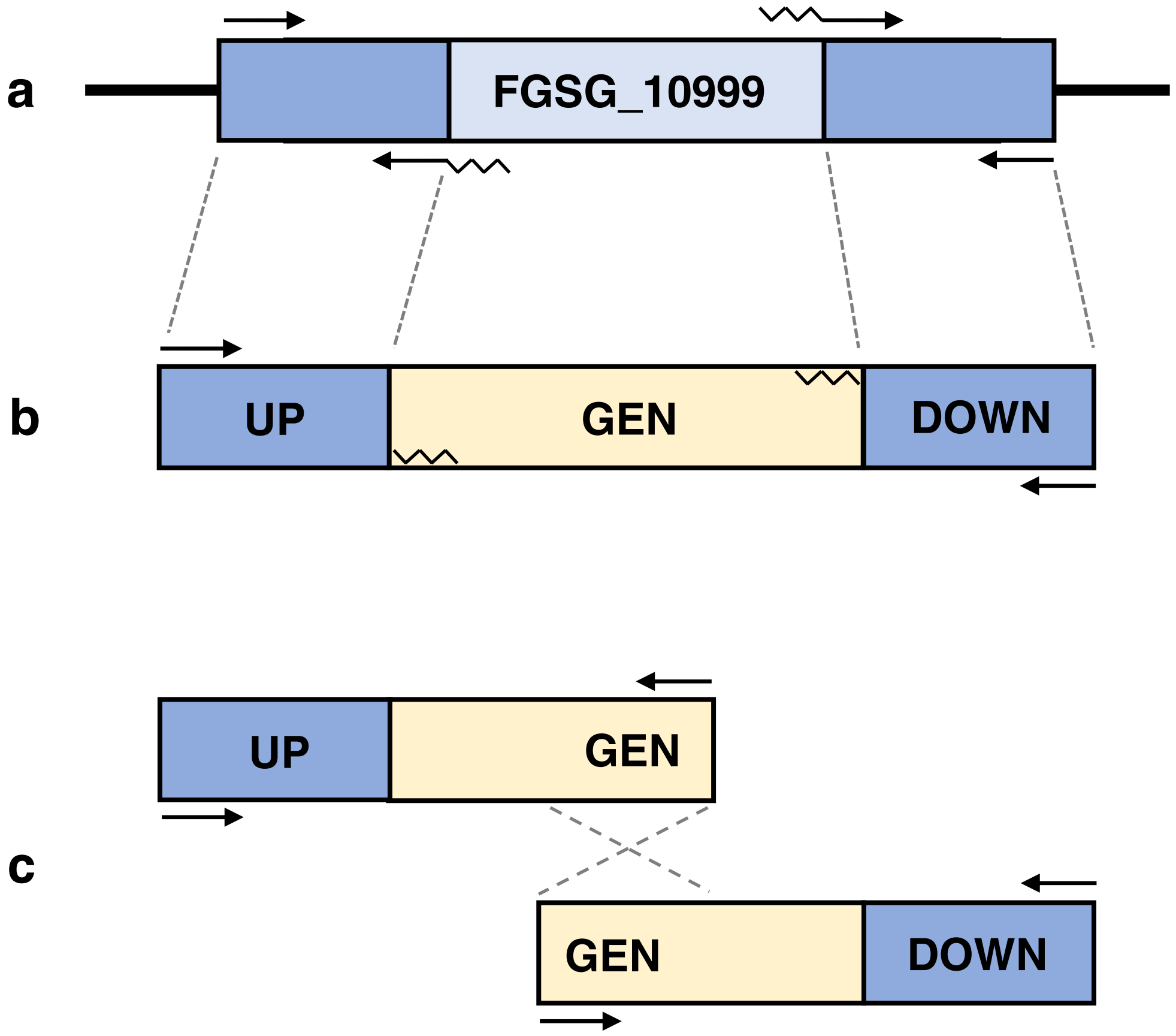


**Figure S11**. Split marker disruption cassette containing the geneticin resistance gene (*Gen*) used for replacing the *Fusarium graminearum FGSG_10999* (*xylA*) gene in both the wild-type (PH1 strain) and the ΔxylB mutant strain. (a) Amplification of the flanking homology regions. (b) Fusion of the homologous flanking regions to the antibiotic resistance marker gene. (c) Amplification of the split marker constructs 10999_SPLIT1_Gen (5') and 10999_SPLIT2_Gen (3') using nested primers.
