# Supplementary material for: Deletion of Major Endoxylanase Genes in Fusarium graminearum Uncovers Distinct Virulence Phenotypes Influenced by the Genetic Background
Source: Mol Plant Pathol. 2026 Oct 27;27(10):e70362. doi: 10.1111/mpp.70362 (PMC13714102; doi:10.1111/mpp.70362)
Supplement: Supplementary file 9 [file MPP-27-e70362-s009.pdf]

FIGURE S7

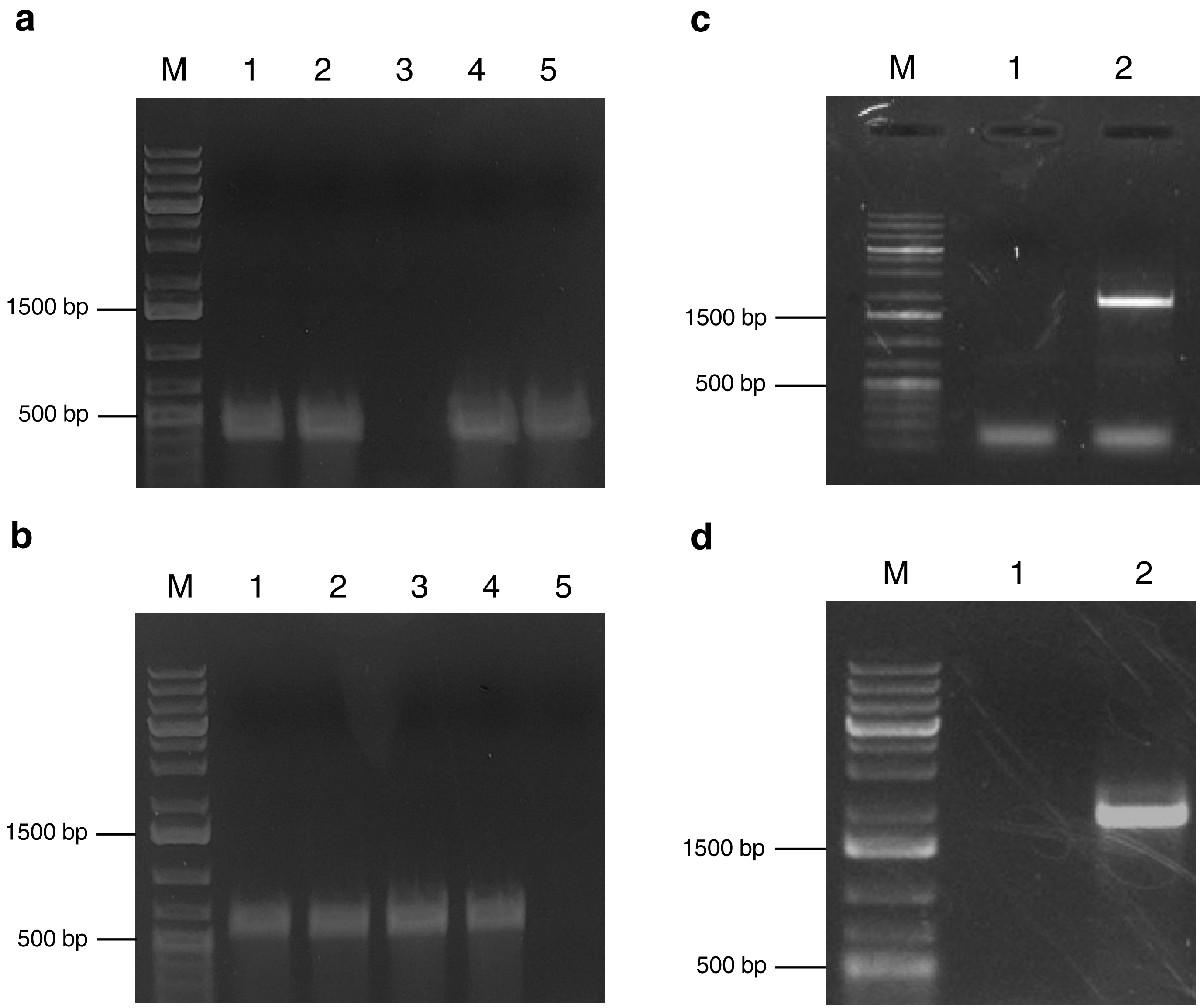

Figure S7. PCR screening of double knock-out (KO) mutant obtained using the ΔxylA_3F strain as genetic background. Primers internal to the *xylB* gene (a) and to the hygromycin resistance gene (b) were used to screen putative mutants. Three ectopic mutants (lanes 1, 2 and 4; a-b) and one double KO mutant, named ΔΔxylA+B (lane 3; a-b), were obtained. Amplification of the of the UP (c) and DOWN (d) regions flanking the cassette used to obtain the KO mutants confirms homologous recombination in the ΔΔxylA+B double KO mutant. (a-b) Lanes 1, 2 and 4: ectopic mutants; lane 3: ΔΔxylA+B double KO mutant; lane 5: *F. graminearum* wild-type (WT; PH1strain). (c-d) Lane 1: WT (PH1 strain); lane 2: ΔΔxylA+B mutant strain; M = marker 1Kb+ (ThermoScientific).
