# Supplementary material for: Deletion of Major Endoxylanase Genes in Fusarium graminearum Uncovers Distinct Virulence Phenotypes Influenced by the Genetic Background
Source: Mol Plant Pathol. 2026 Oct 27;27(10):e70362. doi: 10.1111/mpp.70362 (PMC13714102; doi:10.1111/mpp.70362)
Supplement: Supplementary file 10 [file MPP-27-e70362-s010.pdf]

FIGURE S4

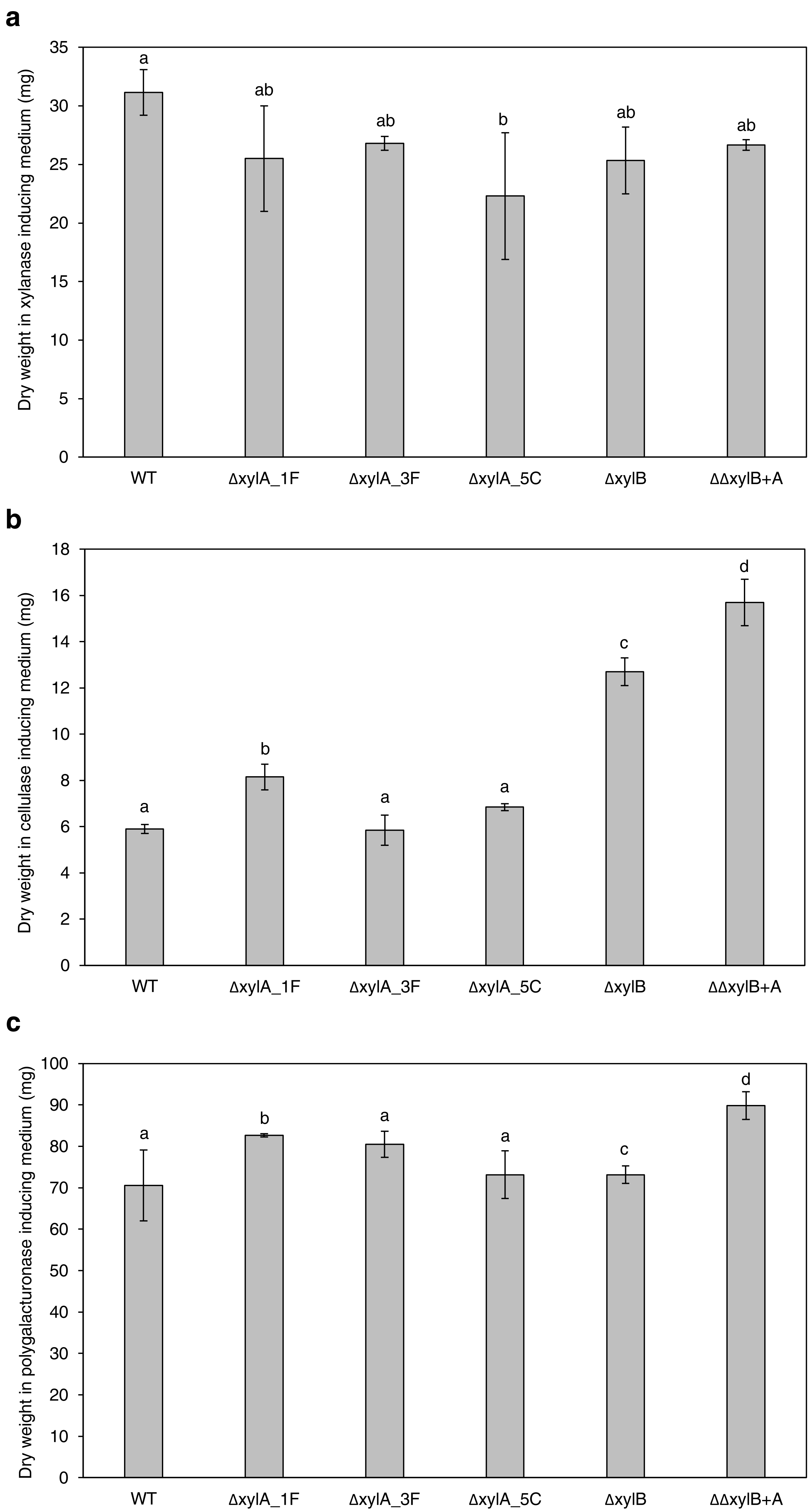

Figure S4. Mycelia dry weight produced by wild-type (WT) and mutant strains in xylanase (a), cellulase (b) and polygalacturonase (c) inducing media. Conidia (1 x 10^4 mL^{-1}) were grown in xylan-, carboxymethyl cellulose (CMC)- or pectin- containing Szécsi liquid media for 4 days. Mycelia were then filtered, washed twice with sterile water, and dry hooved at 80°C for 4 days. Data are presented as the average of three independent experiments for each inducing medium. Bars indicate standard deviation. Statistical analyses were conducted using one-way ANOVA followed by Tukey-Kramer post hoc test. Different letters indicate significant differences at $p < 0.05$.
