# Supplementary material for: Deletion of Major Endoxylanase Genes in Fusarium graminearum Uncovers Distinct Virulence Phenotypes Influenced by the Genetic Background
Source: Mol Plant Pathol. 2026 Oct 27;27(10):e70362. doi: 10.1111/mpp.70362 (PMC13714102; doi:10.1111/mpp.70362)
Supplement: Supplementary file 11 [file MPP-27-e70362-s012.pdf]

FIGURE S8

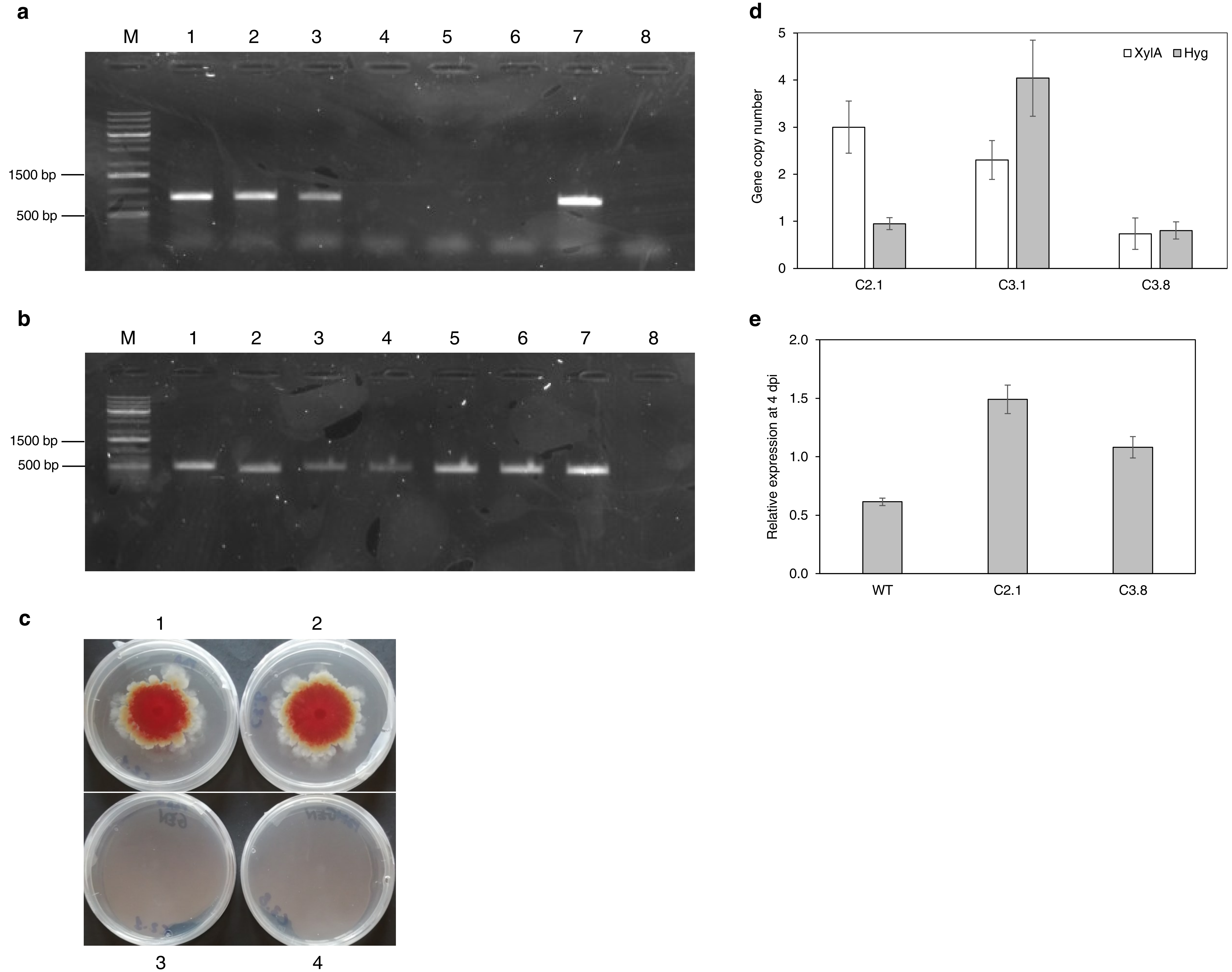

Figure S8. (a, b) PCR screening of the complemented strains obtained from the ∆xylA_3F mutant. Primers amplifying the *Fusarium graminearum XylA* full open reading frame (a) and the hygromicin resistance gene (b) were used to screen putative complemented strains. Lane 1: C2.1; lane 2: C3.1; lane 3: C3.8; lane 4: C4.3; lane 5: C4.4; lane 6: C4.5; lane 7: C4.6; lane 8: no template control. M = marker 1Kb+ (ThermoScientific). (c) Growth of C2.1 and C3.8 in potato dextrose agar (PDA) supplemented or not with 100 µg/ml geneticin (GEN). 1: C2.1 in PDA; 2: C3.8 in PDA; 3: C2.1 in PDA+GEN; 4: C3.8 in PDA+GEN. (d) Quantification of XylA and hygromycin copy number in the genome of the complemented strains. Quantification was carried out by qPCR following the protocol described by Solomon et al. (2008) and using the *Fusarium graminearum* translation elongation factor 1-alpha (TEF1), present in a single copy in the fungal genome, as a reference gene. Relative expression levels were analyzed using the Rotor-Gene v. 2.0.3.2 software (Qiagen). Data represent the mean ± standard deviation of three replicates. (e) Expression analysis of *Fusarium graminearum* XylA gene. Total RNA was extracted from mycelia of WT, C2.1 and C3.8 complemented strains collected after 4 days of growth in carboxymethyl cellulose (CMC) liquid medium. RTqPCR was performed using the *F. graminearum* β-tubulin gene as housekeeping. Relative expression levels were analyzed using the Rotor-Gene v. 2.0.3.2 software (Qiagen). Data represent the mean of at least three replicates. Error bars indicate standard deviation.
