# Supplementary material for: Deletion of Major Endoxylanase Genes in Fusarium graminearum Uncovers Distinct Virulence Phenotypes Influenced by the Genetic Background
Source: Mol Plant Pathol. 2026 Oct 27;27(10):e70362. doi: 10.1111/mpp.70362 (PMC13714102; doi:10.1111/mpp.70362)
Supplement: Supplementary file 12 [file MPP-27-e70362-s013.pdf]

FIGURE S3

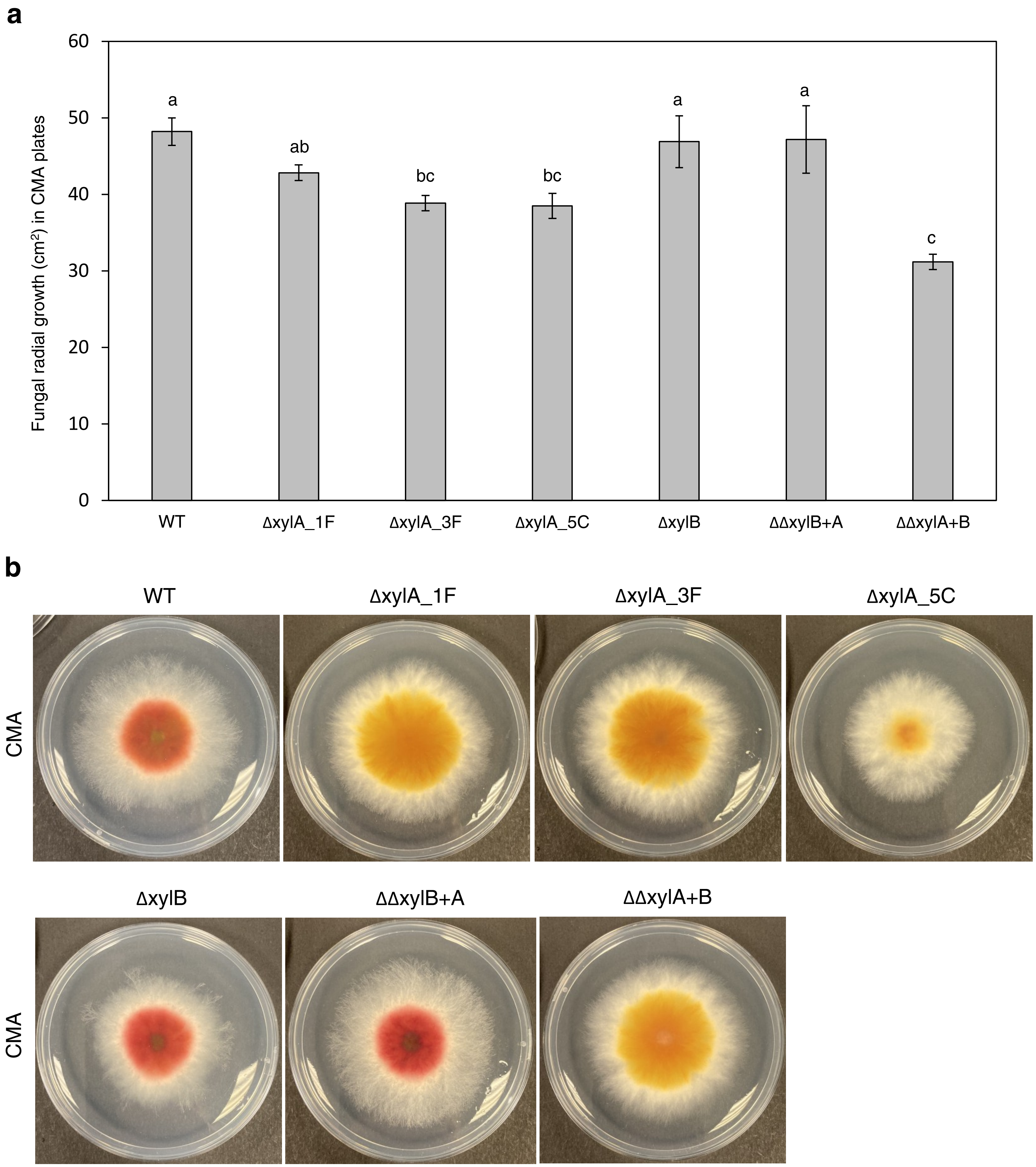

Figure S3. Vegetative growth of *F. graminearum* wild-type (WT) and xylanase mutant strains on complete medium agar (CMA). (a) Radial growth was measured after 7 days at 25 °C and expressed in cm^2 as the average of at least three independent replicates per strain. Bars indicate standard errors. Statistical analyses were conducted using one-way ANOVA followed by Tukey-Kramer post hoc test. Different letters indicate significant differences at $p < 0.05$. (b) Mycelia growth in CMA plates photographed at 7 dpi.
