# Supplementary material for: Deletion of Major Endoxylanase Genes in Fusarium graminearum Uncovers Distinct Virulence Phenotypes Influenced by the Genetic Background
Source: Mol Plant Pathol. 2026 Oct 27;27(10):e70362. doi: 10.1111/mpp.70362 (PMC13714102; doi:10.1111/mpp.70362)
Supplement: Supplementary file 13 [file MPP-27-e70362-s011.docx]

**Table S1**. Primers used in the present study.

| Primer name | Primer sequence (5’-3’) | Size (bp) |
| --- | --- | --- |
| Primers to produce the *FGSG_10999* (*xylA*) gene disruption construct (tail sequences underlined) | | |
| FG_10999UP_for | CAACCCGACCGAATCTCTA | 999 |
| FG_10999UP_rev | AACAACTGGCATGAATTCATCGATGATATCAGATCAAGGGCGCGAGAGTAGTTCT |  |
| FG_10999DWN_for | GATCCTCTAGAGTCGACCTGCAGGCATGCATGTCAGCTGTGGTCGGTTAC | 854 |
| FG_10999DWN_rev | GACACGCCTAGTTCGGACAT |  |
| SplitUp_GEN_for | TGAGATGAAAATCCGGGAAG | 2711 |
| SplitUp_GEN_rev | AATATCACGGGTAGCCAACG |  |
| SplitDown_GEN_for | GAAAGCGCGTTGGATTAGAG | 2718 |
| SplitDown_GEN_rev | GTGGCATTGACAAGGTAGGG |  |
| Primers for the preliminary screening of the ΔxylA, ΔΔ and complemented mutants | | |
| FG_10999_int_F  FG_10999_int_R | GGCGCAACACTGGTAACTTT  AGATGAGCCACTGCTCTGGT | 543 |
| Gen_For | AGGATCTCCTGTCATCTCA | 400 |
| Gen_Rev | CCAAGCTCTTCAGCAATA |  |
| FG_03624_int_F | GCACCTACAACCCCTCTTCC | 455 |
| FG_03624_int_R | CTGCGTGAAGTCCAGCTACA |  |
| HygInt_For | CCCCAATGTCAAGCACTTCC | 553 |
| HygInt_Rev | AGCCCGATTTCCATTCCTCA |  |
| XylAcompl-F | ATGGTCTCGTTCAAATCC | 802 |
| XylAcompl-R | TTAACTAGTCTGGACATAGATAGA |  |
| Primers for the second screening of the ΔxylA and ΔΔ mutants | | |
| 10999OUTUP_for | ATCGGGCTAGATGTGTGTCG | 1320 |
| 10999OUTUP_rev | TGACATCCTCAACGCCAGAC |  |
| 10999OUTDOWN_for | CCCCTGGGTTCGCAAAGATA | 1350 |
| 10999OUTDOWN_rev | TGAGAGTACTGCACGCATCG |  |
| 03624UP_Check_for | AGTTGCTGCTGGTTTCTTGG | 1896 |
| 03624UP_Check_rev | GGCTCCAACAATGTCCTGAC |  |
| 03624DN_Check_for | TTGCCGTCTGTGGTATCTGT | 1887 |
| 03624DN_Check_rev | CGGGGAAGTTATTGTGCGAG |  |
| Primers for quantification of geneticin, hygromycin and XylA copy number | | |
| FgEF1_for | GCTTTCACCGACTACCCTC | 196 |
| FgEF1_rev | ACCAGAGTCGTAAGATCCCA |  |
| qGen_for | CCTGTCATCTCACCTTGCTC | 178 |
| qGen_rev | GCTCTTCGTCCAGATCATCC |  |
| qHyg_for | GAATTGAGGAATGGAAATCGGG | 237 |
| qHyg_rev | GCAAAGGGTGTAATTGGGTC |  |
| 10999RTfor | GTCTCGTTCAAATCCCTTCTC | 262 |
| 10999RTrev | TTCCACCGACAAAGTTACCA |  |
| Primers for RT-qPCR gene expression analysis in planta | | |
| TaActF | TCCTGTGTTGCTGACTGAGG | 230 |
| TaActR | GGTCCAAACGAAGGATAGCA |  |
| Tri6_10F | TCTTTGTGAGCGGACGGGACTTTA | 245 |
| Tri6_4R | ATCTCGCATGTTATCCACCCTGCT |  |
| betaTubF1 | ACTTCCGTCTGTTCCGTG | 171 |
| betaTubR1 | TTCCATCTCGTCCATACCCT |  |
| FGSG_01831_for | GCCTACATCCCTTGCAGTGG | 190 |
| FGSG_01831_rev | GGATGCCTTGGTCAAGGATGG |  |
| FGSG_02326_for | GTCCCTCATTGTTGACATTGGAGG | 150 |
| FGSG_02326_rev | ATCGGTGCCAATCACATGCT |  |
| FGSG_03600_for | TACCGGTGGTAGCTACTCGT | 193 |
| FGSG_03600_rev | GGTGTTGGCAATCTTCTCCAAC |  |
| FGSG_06616_for | AACTGGCACACCAACCAGAA | 166 |
| FGSG_06616_rev | TGTCCTCACTAGGGTCCTCG |  |
| FGSG_08079_for | TCCCGTGTTTGGCTGAACTATC | 200 |
| FGSG_08079_rev | GGCTTGAGTTCAGGCAATGC |  |
| FGSG_10212_for | CGGCCACCACTGTCTCCTA | 145 |
| FGSG_10212_rev | CCACCGATGTACTTGGTGGG |  |
| 10999RTfor | GTCTCGTTCAAATCCCTTCTC | 262 |
| 10999RTrev | TTCCACCGACAAAGTTACCA |  |
| Primers for complementing the ΔxylA mutant | | |
| XylA_compl4_F | ATGTATCCAACCCGACCGA | 2777 |
| XylA_compl4_R | CTTGTCCATTCCGAAGCCC |  |
| HygFULL-for | GGTCGGCATCTACTCTATTCCT | 1716 |
| HygFULL-rev | TTGACTAGTGTACCACTTGACCA |  |
